# *Lachnospiraceae bacterium* alleviates alcohol-associated liver disease by enhancing N-acetyl-glutamic acid levels and inhibiting ferroptosis through the KEAP1-NRF2 pathway

**DOI:** 10.1080/19490976.2025.2517821

**Published:** 2025-06-13

**Authors:** Hejiao Zhang, Qiang Hu, Yong Zhang, Lei Yang, Shanfei Tian, Xinru Zhang, Haiyuan Shen, Hang Shu, Linxi Xie, Dongqing Wu, Liangliang Zhou, Xiaoli Wei, Chen Cheng, Jiali Jiang, Hua Wang, Cailiang Shen, Derun Kong, Long Xu

**Affiliations:** a Department of Gastroenterology, The First Affiliated Hospital of Anhui Medical University, Hefei, China; b Department of Orthopedics and Spine Surgery, The First Affiliated Hospital of Anhui Medical University, Hefei, China; c School of Basic Medical Sciences, Anhui Medical University, Hefei, Anhui, China; d Department of Oncology, The First Affiliated Hospital of Anhui Medical University, Hefei, China; e Inflammation and Immune Mediated Diseases Laboratory of Anhui Province, Anhui Medical University, Hefei, China; f Department of Infectious Diseases, The Second Affiliated Hospital of Anhui Medical University, Hefei, China

**Keywords:** Alcohol-associated liver disease, *Lachnospiraceae bacterium*, N-Acetyl-glutamic acid, NRF2, ferroptosis

## Abstract

Alcohol-associated liver disease (ALD) is a prevalent global health issue primarily caused by excessive alcohol consumption. Recent studies have highlighted the gut-liver axis’s protective role against ALD, mainly through gut microbiota. However, the precise mechanism remains ill-defined. Our results showed a significant reduction in *Lachnospiraceae bacterium* in the gut microbiota of ALD patients and ethanol (EtOH)-fed mice, as revealed by 16S rDNA sequencing. Supplementation with *Lachnospiraceae bacterium* strains in mice significantly reduced inflammation, hepatic neutrophil infiltration, oxidative stress, and improved gut microbiota and intestinal permeability. Multi-omics analysis identified N-Acetyl-glutamic acid (NAG) as the most significantly altered metabolite following *Lachnospiraceae bacterium* supplementation, with levels positively correlated to *Lachnospiraceae bacterium* colonization. NAG treatment exhibited significant protective effects in EtOH-exposed hepatocyte cell lines and EtOH-fed mice. Mechanistically, NAG confers hepatoprotection against ALD by activating the KEAP1-NRF2 pathway, inhibiting ferroptosis. Notably, the protective effects of NAG were reversed by the NRF2 inhibitor. In conclusion, oral supplementation with *Lachnospiraceae bacterium* mitigates alcohol-induced liver damage both *in vivo* and *in vitro* by inhibiting ferroptosis through NAG-mediated activation of the KEAP1-NRF2 pathway. *Lachnospiraceae bacterium* may serve as promising probiotics for future clinical applications.

## GRAPHICAL ABSTRACT

**Figure:**
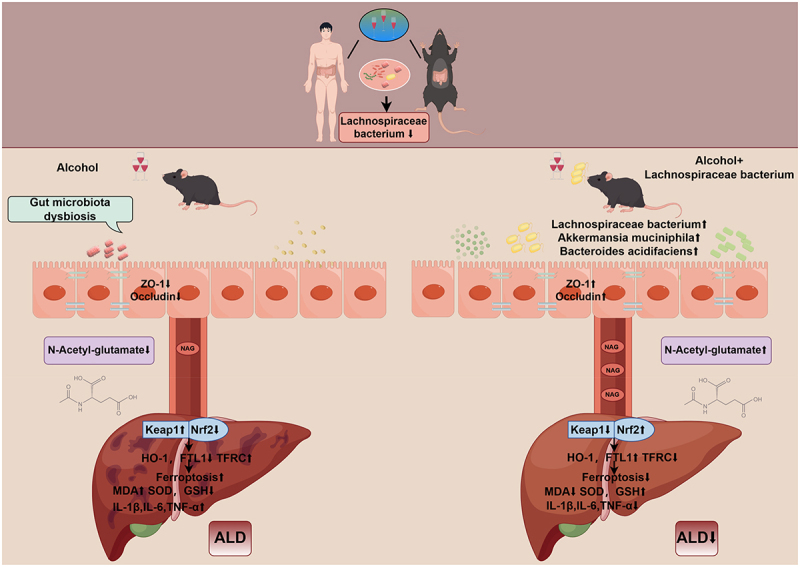

## Introduction

In recent years, alcoholism has emerged as a global medical concern with significant social and economic repercussions.^1,2^ Alcohol-associated liver disease (ALD) is one of the most prevalent liver diseases worldwide and is primarily caused by excessive alcohol consumption. ALD initially manifests as alcohol-associated liver injury and progresses to alcohol-associated hepatitis, alcohol-associated fibrosis, cirrhosis, and hepatocellular carcinoma.^3^ Each stage of ALD poses a substantial public health burden.^4,5^ Despite its high prevalence, effective treatments for ALD remain limited, underscoring the urgent need for novel therapeutic strategies.

The pathogenesis of ALD is multifaceted and involves environmental factors, genetic susceptibility, immune responses, and the gut microbiota. The integrity of the intestinal epithelial barrier, the composition of the intestinal microbiome, and continuous surveillance by the intestinal immune system collectively minimize the translocation of bacterial products to the liver via the portal circulation.^6^ Chronic ethanol consumption disrupts the intestinal microbiome, characterized by a decreased abundance of Bacteroidetes phyla and increased levels of Actinobacterium and Proteobacteria phyla in mice.^7^ In addition, alcohol impairs intestinal permeability and barrier function, making it easier for harmful substances to leak into the liver and circulatory system. Recent research has highlighted the deleterious effects of alcohol on the gut-liver axis, emphasizing the influence of the gut microbiota on liver disease development.

Numerous studies have demonstrated the potential of probiotics in mitigating ALD in animal models. Prominent examples include *Lactobacillus acidophilus*, *Lactobacillus rhamnosus*, and *Bifidobacterium bifidum*.^8–10^ Meanwhile, Lachnospiraceae has been identified as having protective effects in various diseases. Metagenomic studies estimate that Lachnospiraceae constitute 20–40% of bacteria found in the feces of healthy individuals.^11^ Members of the Lachnospiraceae family are anaerobic bacteria belonging to the Clostridia class and hold potential for applications in bioeconomics and enteric health treatments.^12^ They produce favorable metabolites, such as acetate, propionate, and butyrate.^13,14^ Several studies have documented the protective effects of Lachnospiraceae in various disease contexts. For example, *Blautia wexlerae*, a Lachnospiraceae species, was shown to mitigate obesity and diabetes induced by a high-fat diet by modulating gut microbiota composition in mice.^15^ Similarly, *Blautia producta* alleviated hyperlipidemia in mice by activating G protein-coupled receptor 120 through the metabolite 12-methylmyristic acid.^16^ However, the role of Lachnospiraceae species in ALD remains insufficiently explored.

Ferroptosis, a novel form of non-apoptotic programmed cell death, is characterized by iron accumulation and lipid peroxidation.^17^ Inhibition of ferroptosis has shown promise in alleviating ALD, non-alcohol-associated fatty liver disease, and hepatic ischemia-reperfusion injury (HIRI).^18–20^ For instance, Wang et al. reported that metformin remodeled the gut microbiota, increased gamma-aminobutyric acid levels, and conferred resistance to HIRI-induced ferroptosis.^21^ Yang et al. demonstrated that butyrate attenuates acute liver injury-induced ferroptosis by activating the AMPK-P62-NRF2 signaling pathway.^22^ These studies highlight the significance of NRF2 as a critical negative regulator of ferroptosis.

In the current study, we found a decreased abundance of *Lachnospiraceae bacterium* in patients with ALD and EtOH-fed mice, as revealed by 16S rDNA sequencing. We hypothesized that *Lachnospiraceae bacterium* might have a protective effect against ALD. To test this hypothesis, we employed a mouse model of ALD supplemented with *Lachnospiraceae bacterium* to evaluate its therapeutic effects. Furthermore, proteomics and metabolomics analyses were conducted to elucidate the underlying mechanisms, providing a foundation for novel therapeutic strategies for ALD treatment.

## Materials and methods

### Study population

This prospective cohort study was conducted from 2021 to 2024 at the Department of Gastroenterology and Physical Examination Center of the First Affiliated Hospital of the Anhui Medical University (Table 1). Given the high prevalence of alcohol consumption among Chinese males, only male participants were included. A total of 40 subjects were recruited, comprising 20 patients with alcohol-associated liver disease (ALD) and 20 healthy controls (HC). ALD patients were categorized into subgroups: alcohol-associated fatty liver disease (AFL, *n* = 4), alcohol-associated hepatitis (AH, *n* = 4), severe alcohol-associated hepatitis (SAH, *n* = 1), and alcohol-associated cirrhosis (ALC, *n* = 11), based on previously published diagnostic criteria.^23,24^ Inclusion criteria^1^: History of alcohol consumption exceeding 40 g/day for over 1 year.^2^ Confirmed disease diagnosis via imaging or liver biopsy. Exclusion criteria^1^: Age under 18 or over 75 years.^2^ Recent use of oral antibiotics, probiotics, or medications within two weeks.^3^ Comorbid severe cardiovascular or cerebrovascular diseases, viral liver diseases, immune-mediated liver injury, or hepatocellular carcinoma.^4^ Psychiatric illnesses. HC participants underwent physical examinations to confirm no history of metabolic disorders, infectious diseases, or cancer. Fasting blood samples were collected upon admission, and fecal samples were obtained from the first bowel movement. Fecal samples were stored in sterile containers at −80°C.

Ethical approval was obtained from the First Affiliated Hospital of the Anhui Medical University Ethics Committee (PJ-2024–09–46), and all the participants provided written informed consent.

### Mouse experiments

Ten-week-old female C57BL/6J mice were used in a chronic and binge ethanol feeding model (NIAAA model) with 6–8 mice per group. Mice received 200 μL of *Lachnospiraceae bacterium* (5 × 10^8^ CFU) or PBS via gavage every other day during ethanol or control liquid diet feeding. A 0.1% NAG solution (MACKLIN, Shanghai, China) was added to the diets. For ML385 (MCE, New Jersey, USA) treatment, mice were intraperitoneally injected with 30 mg/kg body weight (dissolved in DMSO) every other day. Food intake and body weight were monitored daily. On the final day, mice were gavaged at 8 a.m., and serum, liver, ileum, and cecum contents were collected 9 hours later for analysis.

### Bacterial preparation

*Lachnospiraceae bacterium* (ATCC BAA-2278) was cultured under anaerobic conditions at 37°C. Bacterial cells were harvested by centrifugation (6,000 × g, 10 min) and resuspended in 200 μL of PBS at a 5 × 10^8^ CFU concentration.

### Biochemical assays

#### Serum ALT, AST, and LPS

Alanine aminotransferase (ALT) and aspartate aminotransferase (AST) levels were measured using an automatic biochemical analyzer (Myriad, Shenzhen, China). Serum LPS concentrations were determined by ELISA (ELK Biotechnology, Wuhan, China).

#### Liver tissue assays

Liver triglycerides (TG), malondialdehyde (MDA), superoxide dismutase (SOD), and glutathione (GSH) were assessed in 10% liver homogenates using Jiancheng kits (Nanjing, China).

#### Intestinal permeability

Mice were gavaged with 200 µL of 80 mg/mL FITC-dextran (4 kDa, TargetMol, Boston, USA) 5 hours post-ethanol gavage. Blood samples were collected 4 hours later, diluted 1:1 in PBS, and fluorescence was measured (excitation: 485 nm, emission: 530 nm).

#### Ferrous ion content

Liver ferrous ion levels were measured using an assay kit (Elabscience, Wuhan, China). Liver tissue (200 mg) was lysed and centrifuged (15,000 × g, 10 min), and absorbance was measured at 593 nm.

#### ROS assay

Intracellular ROS levels were measured using dichlorodihydrofluorescein diacetate probe. AML12 cells were incubated with the probe in serum-free DMEM/F12 medium, and fluorescence intensity was analyzed by flow cytometry (BD Biosciences).

### Flow cytometry

Liver mononuclear cells were stained with antibodies (APC-CY7 anti-CD45, FITC anti-CD11b, PerCP-Cyanine5.5 anti-Ly6G, and APC anti-Ly6G) from BioLegend. Samples were analyzed using a BD FACS Celesta flow cytometer and FlowJo v10.8 software.

### Histology

Liver tissue was fixed, paraffin-embedded, and stained with hematoxylin and eosin (H&E) for injury assessment. Hepatic steatosis was evaluated using oil red O on OCT-embedded sections (6 μm).

### Western blotting

Proteins were extracted from liver and ileum tissues or AML12 cells using RIPA lysis buffer (Beyotime, Shanghai, China) containing protease and phosphatase inhibitors. SDS-PAGE separated proteins, transferred to PVDF membranes, and probed with the following antibodies: anti-KEAP1 (1:1000, Proteintech, China), anti-NRF2 (1:1000, Proteintech, China/1:1000, Cell Signaling Technology, USA), anti-HO-1 (1:1000, Proteintech, China), anti-FTL1 (1:1000, Proteintech, China), anti-TFRC (1:1000, Proteintech, China), anti-ZO-1 (1:1000, Affinity, China), anti-Occludin (1:1000, Invitrogen, USA), and anti-β-actin (1:20,000, Proteintech, China).

### Quantitative real-time polymerase chain reaction (qRT-PCR)

Total RNA was extracted using TRIZOL reagent (Takara, Tokyo, Japan). Reverse transcription was performed using the Vazyme RT III Kit (Vazyme, Nanjing, China). Gene expression levels were quantified using SYBR Green reagent (Vazyme, Nanjing, China) on a real-time PCR system (Thermo Fisher Scientific, USA). GAPDH or 16s mRNA levels were used for normalization. Primer sequences are listed in Table 2.

**Table 1.** Characteristics and liver parameters of healthy controls (HC) and patients with alcohol-associated liver disease (ALD).

Parameters	HC	ALD
Sex (male/female)	20/0	20/0
Age (years)	51.65 ± 1.569	50.35 ± 11.70
Body mass index (kg/m ^2^)	22.13 ± 1.569	22.25 ± 1.588
Alcohol consumption (years)	0	22.05 ± 7.577
ALT(U/L)	13.80 ± 4.797	91.40 ± 41.84
AST(U/L)	17.75 ± 5.056	176.1 ± 95.04
AST/ALT	1.333 ± 0.2563	1.875 ± 0.1455
GGT(U/L)	24.2 ± 5.672	286.4 ± 160.0

Data are shown as the Mean ± SD.

**Table 2.** Sequences of primers for qPCR.

Genes name	Forward primer	Reverse primer
*GAPDH*	AGCAGCCGCATCTTCTTGTGCAGTG	GGCCTTGACTGTGCCGTTGAATTT
*IL-1β*	TCGCTCAGGGTCACAAGAAA	CATCAGAGGCAAGGAGGAAAAC
*TNF-α*	AGGCTGCCCCGACTACGT	GACTTTCTCCTGGTATGAGATAGCAAA
*IL-6*	TCCATCCAGTTGCCTTCTTG	TTCCACGATTTCCCAGAGAAC
*KEAP1*	GATATGAGCCAGAGCGGGAC	CATACAGCAAGCGGTTGAGC
*NRF2*	AAAATCATTAACCTCCCTGTTGAT	CGGCGACTTTATTCTTACCTCTC
*HMOX1*	CAAGCCGAGAATGCTGAGTTCATG	CAAGCCGAGAATGCTGAGTTCATG
*FTL1*	ATGGGCAACCATCTGACCAA	TTGAGAGTGAGGCGCTCAAA
*TFRC*	AAGTGACGTAGATCCAGAGGG	GACAATGGTTCCCCACCAAA
*GCLC*	CTGCACATCTACCACGCAGT	TTCATGATCGAAGGACACCA
*ACSL4*	CCTTTGGCTCATGTGCTGGAACT	CAGCGGCCATAAGTGTGGGTTT
*LPCAT3*	GGCCTCTCAATTGCTTATTTCA	AGCACGACACATAGCAAGGA
*16s*	CCGTCAATTCMTTTGAGTTT	ACTCCTACGGGAGGCAGCAG
*Lb*	AGAGTTTGATCCTGGCTCAG	GGTTACCTTGTTACGACTT

### Liver proteomics analysis

Fresh mouse liver (200 mg) was homogenized in lysis buffer (8 M urea + 1% SDS) containing protease inhibitor at a ratio of 100:1. Protein concentrations were measured and quantified using a BCA kit. Sample preparation included protein denaturation, reductive alkylation, enzymatic digestion, and desalting of peptides. The desalted lyophilized peptides were redissolved in 0.1% formic acid for analysis. DIA (Data-independent acquisition) data was acquired using a timsTOF Pro2 mass spectrometer (Bruker Daltonics) coupled with an UltiMate 3000 UHPLC system (Thermo Fisher Scientific, USA). Differential protein and KEGG pathway analyses were conducted on the DIA data using Spectronaut18 with the factory default settings.

### LC-MS and GC-MS non-targeted metabolomics analysis

Fresh mouse cecum content was extracted using pre-cooled 80% methanol. After centrifugation, supernatants were separated chromatographically on a Waters ACQUITY UPLC BEH Amide column. Metabolites were detected using an AB SCIEX Triple TOF 6600 mass spectrometer. The data were normalized, standardized, and subjected to multivariate statistical and functional analyses of differential metabolites.

### LC-MS targeted metabolomics analysis

Portal vein serum (50 μL) was mixed with 150 μL pure methanol, vortexed for 5 min, and centrifuged at 13,000 rpm for 10 min at 4°C. The supernatant was analyzed using a Welch Ultimate XB-C8 column (150 × 4.6 mm, 5 μm) at a 0.8 mL/min flow rate. The aqueous phase contained 0.1% formic acid in water, while methanol served as the organic phase. The autosampler was maintained at 10°C, and the injection volume was 5 μL.

Detection was performed in scanning mode using a TSQ Quantum triple quadrupole mass spectrometer (Thermo Fisher Scientific, USA). NAG standard (Sigma Aldrich, USA) solutions were prepared at various concentrations and used as positive controls. Chromatogram acquisition and integration were performed using Xcalibur 3.0 software with linear regression (1/X^2^ as the weighting factor).

### 16S rDNA analysis

Microbial DNA was extracted using a Fecal Genomic DNA Kit from human feces or mouse cecum contents (Magen, Guangzhou, China). DNA concentration and purity were assessed using a NanoDrop 2000 spectrophotometer (Thermo Fisher Scientific, USA) and 1% agarose gel electrophoresis. The V3-V4 region of the 16S rDNA gene was then amplified, and sequencing was performed on the Illumina NovaSeq 6000 platform to generate 250 bp paired-end reads (OE Biotech Company, Shanghai, China). Species abundance profiles were analyzed using STAMP and indicator, while QIIME 2 was used for species diversity analysis.

### CCK-8 assay

AML12 cells (mouse normal liver cell line; Pricella Biotechnology Wuhan, China) were cultured in DMEM/F12 medium with 10% fetal bovine serum and 1% penicillin/streptomycin at 37°C in 5% CO_2_. Cells were pre-incubated with NAG for 2 hours, treated with 300 mm anhydrous ethanol, and incubated for 24, 48, and 72 hours. Cell viability was assayed using the CCK-8 kit (Beyotime, Shanghai, China).

### Colony formation assay

AML12 cells (1000 cells/well) were seeded into 6-well plates and cultured for 14 days. Cells were fixed with 4% paraformaldehyde, stained with crystal violet, washed, air-dried, and photographed.

## Statistical analysis

Data were analyzed using GraphPad Prism 8.30 software. Results were expressed as mean±standard deviation (SD) for normally distributed data. Comparisons between two groups were performed using unpaired t-test. One-way analysis of variance (ANOVA) was applied for comparisons among three or more groups with one independent variable. Statistical significance was defined as *p* < 0.05 (*p < 0.05, **p < 0.01, ***p < 0.001).

## Results

### *The abundance of* Lachnospiraceae bacterium *is significantly reduced in ALD patients and EtOH-fed mice and negatively correlates with liver injury in ALD patients*

**Figure 1.**
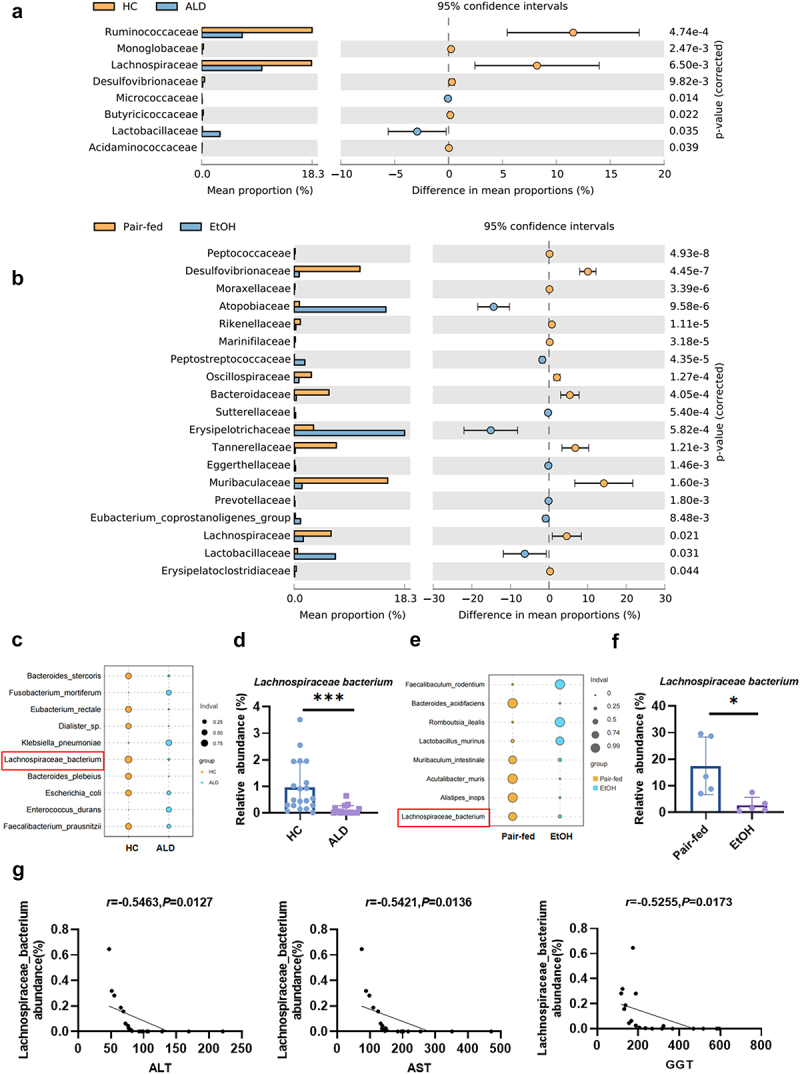
The abundance of *Lachnospiraceae bacterium* is significantly reduced in ALD patients and EtOH-fed mice and negatively correlates with liver injury in ALD patients. (a) The comparison of the relative abundance of gut bacteria between HC and ALD groups at the family level (statistical differences were analyzed using two-sided Welch’s t-test of STAMP). (b) The comparison of the relative abundance of gut bacteria between pair-fed and EtOH groups at the family level (statistical differences were analyzed using two-sided Welch’s t-test of STAMP). (c) Indicator analysis between HC and ALD groups. The bubble plot visualizes biomarkers; indval represents the ratio of specificity to occupancy. (d) Relative abundance of *Lachnospiraceae bacterium* between HC and ALD groups. (e) Indicator analysis between pair-fed and EtOH groups. (f) Relative abundance of *Lachnospiraceae bacterium* between pair-fed and EtOH groups. (g) Correlation analysis of differential species with serum ALT, AST, and GGT levels. *n* = 20 per group. HC: healthy controls; ALD: alcohol-associated liver disease patients. *p < 0.05, **p < 0.01, ***p < 0.001.

To investigate the association between alcohol consumption and the gut microbiota, we collected feces from healthy controls (HC) and patients with ALD. The mean duration of alcohol consumption among ALD patients was 22.05 ± 7.577 years. As expected, serum ALT, AST, and GGT levels were significantly elevated in ALD patients compared to HC (Supplementary Table S1). We further utilized the NIAAA mouse model, involving 10 days of chronic ethanol feeding followed by a single binge ethanol exposure, to collect fecal samples from control and EtOH-fed mice. 16S rDNA sequencing analysis of both human and mouse fecal samples revealed distinct microbiota profiles. Using statistical analysis of metagenomic profiles (STAMP), we observed that ALD patients exhibited a higher relative abundance of Lactobacillaceae and Micrococcaceae at the family level compared to the HC group. In contrast, the relative abundance of Ruminococcaceae, Monoglobaceae, Lachnospiraceae, and Desulfovibrionaceae was lower in the ALD group (Figure 1(a)). In mice, the EtOH group showed an increased relative abundance of Atopobiaceae, Erysipelotrichaceae, and Lactobacillaceae, whereas the relative abundance of Desulfovibrionaceae, Bacteroidaceae, and Lachnospiraceae was reduced compared to the pair-fed group (Figure 1(b)).

Interestingly, a comparative analysis of the human and mouse gut microbiota revealed similar bacterial compositions. For instance, Desulfovibrionaceae and Lachnospiraceae were prevalent in the HC and pair-fed groups, while Lactobacillaceae was predominant in the ALD and EtOH groups. At the species level, *Lachnospiraceae bacterium* was significantly reduced in the ALD/EtOH groups compared to HC/pair-fed groups (Figure 1(c–f)). This reduction was further confirmed by quantitative PCR in ALD patients and EtOH-fed mice (Supplementary Fig. S1A, B). Correlation analysis revealed a strong negative association between the relative abundance of *Lachnospiraceae bacterium* and the serum ALT, AST, and GGT levels in ALD patients (Figure 1(g)). Given that members of the Lachnospiraceae family are known to maintain the gut barrier and produce antioxidants that mitigate oxidative stress in the liver,^14,25^ we hypothesized that *Lachnospiraceae bacterium* plays a protective role in the progression of ALD.

### Lachnospiraceae bacterium *attenuates alcohol-associated steatohepatitis in mice*

**Figure 2.**
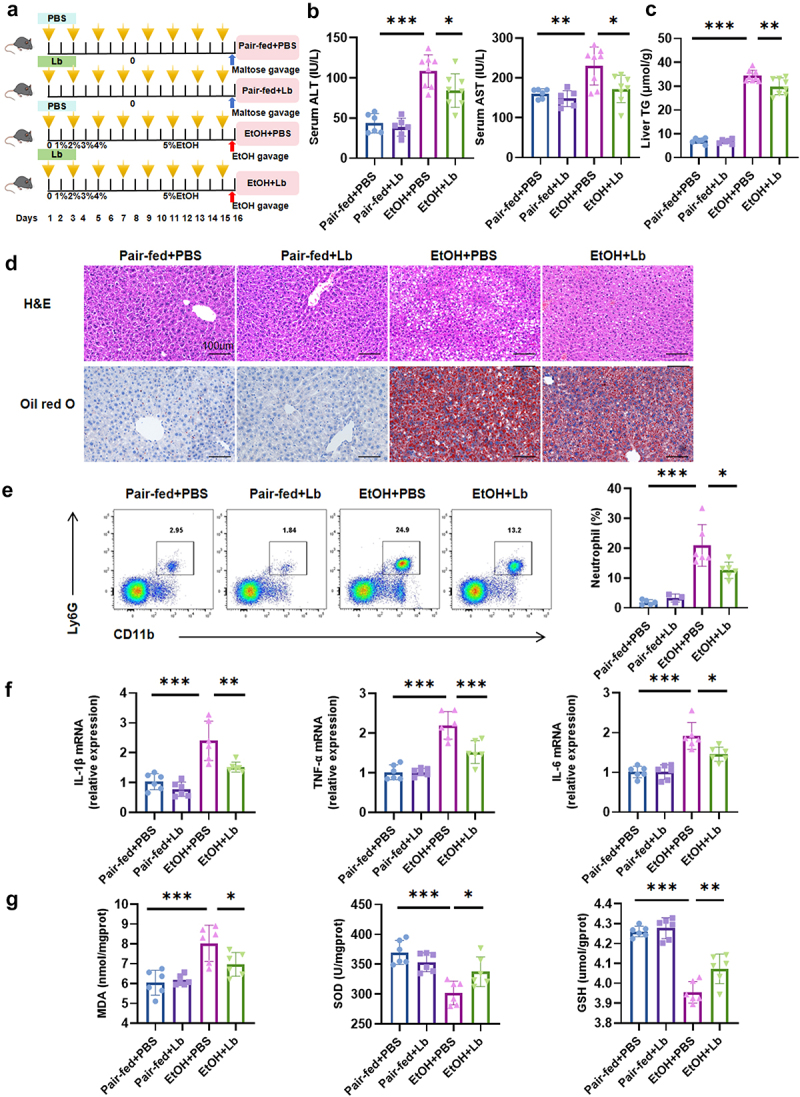
*Lachnospiraceae bacterium* attenuates alcohol-associated steatohepatitis in mice. (a) Schematic of experimental design (yellow arrows indicate gavage with PBS or *Lachnospiraceae bacterium* every other day). (b) Serum ALT and AST levels. (c) Liver TG levels. (d) Representative H&E and Oil red O staining of liver tissue (scale bar: 100 μm). (e) Proportion of neutrophils analyzed by flow cytometry, with corresponding statistical graphs. (f) Hepatic mRNA expression of inflammatory cytokines (*IL-1β, TNF-α, IL-6*). (g) Relative expression of oxidative stress markers (MDA, SOD, GSH) in liver tissue. *n* = 6–8 per group. *p < 0.05, **p < 0.01, ***p < 0.001.

To test the role of *Lachnospiraceae bacterium* in ALD, we administered the bacterium to mice using the NIAAA model via oral gavage every alternate day (Figure 2(a)). Body weight changes and average food intake showed no significant differences across experimental groups (Supplementary Figure 2a, b). Fortunately, successful colonization of *Lachnospiraceae bacterium* in mice was confirmed by quantitative PCR (Supplementary Figure 2c). Mice supplemented with *Lachnospiraceae bacterium* exhibited significantly reduced liver injury after ethanol feeding, as evidenced by decreased serum ALT and AST levels (Figure 2(b)). Furthermore, liver triglyceride (TG) level, and histological analysis, including hematoxylin and eosin (H&E), and oil red O staining, revealed a marked reduction in liver steatosis (Figure 2(c,d)).

EtOH-fed mice developed exacerbated liver inflammation, characterized by increased neutrophil infiltration and elevated hepatic mRNA levels of pro-inflammatory cytokines (*IL-1β*, *TNF-α*, and *IL-6*). However, *Lachnospiraceae bacterium* supplementation significantly attenuated liver inflammation (Figure 2(e,f)). Additionally, EtOH-induced oxidative stress in the liver, as indicated by increased MDA levels and decreased SOD and GSH levels, was ameliorated by *Lachnospiraceae bacterium* treatment (Figure 2(g)). These findings suggest that *Lachnospiraceae bacterium* mitigates alcohol-induced liver injury, steatosis, inflammation, and oxidative stress.

### Lachnospiraceae bacterium *modulates gut microbiota composition and significantly increases metabolite N-Acetyl-glutamic acid*

**Figure 3.**
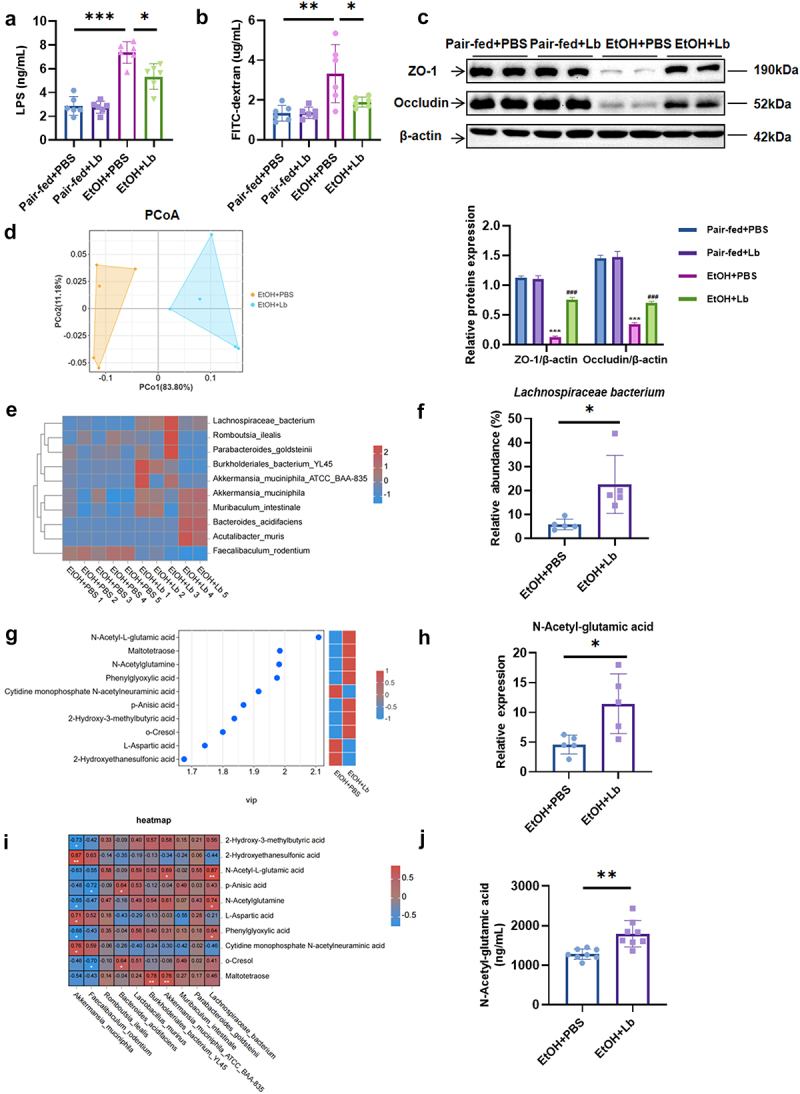
*Lachnospiraceae bacterium* modulates gut microbiota composition and increases metabolite N-Acetyl-glutamic acid. (a) Serum LPS levels. (b) Serum FITC-dextran levels. (c) Protein levels of ZO-1, occludin, and β-actin in ileum tissue with statistical analysis (Pair-fed+PBS vs Pair-fed+Lb vs EtOH+PBS vs EtOH+Lb). (d) PCoA plot of gut microbiota composition (based on Bray-Curtis distances). (e) Heatmap of species-level bacterial composition between EtOH+PBS and EtOH+Lb groups. (f) Histogram showing the relative abundance of *Lachnospiraceae bacterium* between EtOH+PBS and EtOH+Lb groups. (g) VIP plot of differential metabolites distinguishing EtOH+PBS and EtOH+Lb groups. (h) Histogram of NAG levels between EtOH+PBS and EtOH+Lb groups. (i) Heatmap showing Spearman’s correlation between gut microbiota and metabolites. (j) Portal vein serum NAG levels. *n* = 6–8 per group. *p < 0.05, **p < 0.01, ***p < 0.01. For (C): *p < 0.05, **p < 0.01 and ***p < 0.001 vs Pair-fed+PBS, ^#^*p* < 0.05, ^##^*p* < 0.01, ^###^*p* < 0.001 vs EtOH+PBS.

Chronic alcohol consumption disrupts intestinal permeability by impairing intestinal barrier function and altering microbiota composition.^26^ Our data showed that *Lachnospiraceae bacterium* restored intestinal barrier integrity by upregulating tight junction proteins ZO-1 and Occludin while reducing levels of LPS and FITC-dextran, which are indicative of compromised intestinal permeability (Figure 3(a-c)). To further examine the effects of *Lachnospiraceae bacterium* on gut microbiota composition, we performed 16S rDNA sequencing on fecal samples. Principal coordinates analysis (PCoA) revealed significant separation between the gut microbiota of EtOH-fed mice and those treated with *Lachnospiraceae bacterium*, indicating a distinct microbiota structure between the two groups (Figure 3(d)). At the species level, we observed an enrichment of beneficial bacteria, including *Akkermansia muciniphila*, *Bacteroides acidifaciens*, and *Akkermansia muciniphila ATCC BAA-83*, following *Lachnospiraceae bacterium* colonization (Figure 3(e-f)).

Microbial metabolites are integral to maintaining gut homeostasis by providing energy, orchestrating immune responses, and preserving the intestinal barrier integrity.^27,28^ To identify the key metabolites driving the protective effects of *Lachnospiraceae bacterium*, we conducted untargeted metabolomics analyses using LC-MS and GC-MS. The LC-MS analysis revealed increased levels of several metabolites such as N-Acetyl-L-glutamic acid (NAG), maltotetraose, and N-Acetylglutamine in the EtOH+Lb group compared to the EtOH+PBS group. In contrast, cytidine monophosphate N-acetylneuraminic acid, L-Aspartic acid, and 2-Hydroxyethanesulfonic acid were reduced. Among these, NAG exhibited the most pronounced increase (Figure 3(g,h)). Correlation analysis between differential bacterial species and metabolites demonstrated a strong positive association between *Lachnospiraceae bacterium* abundance and metabolites such as NAG, N-Acetylglutamine, and phenylglyoxylic acid, with the strongest correlation observed for NAG (Figure 3I).

GC-MS metabolomics further confirmed the significant elevation of NAG in the EtOH+Lb group, corroborating its positive correlation with *Lachnospiraceae bacterium* abundance (Supplementary Figure 3 A-C). Targeted metabolomics analysis revealed significantly higher levels of portal vein serum NAG in EtOH-fed mice supplemented with *Lachnospiraceae bacterium* (Figure 3J). These findings suggest that NAG is a key metabolite produced by *Lachnospiraceae bacterium* and may play a critical role in its protective effects.

### NAG offers significant protection against EtOH-induced damage in vivo and in vitro

**Figure 4.**
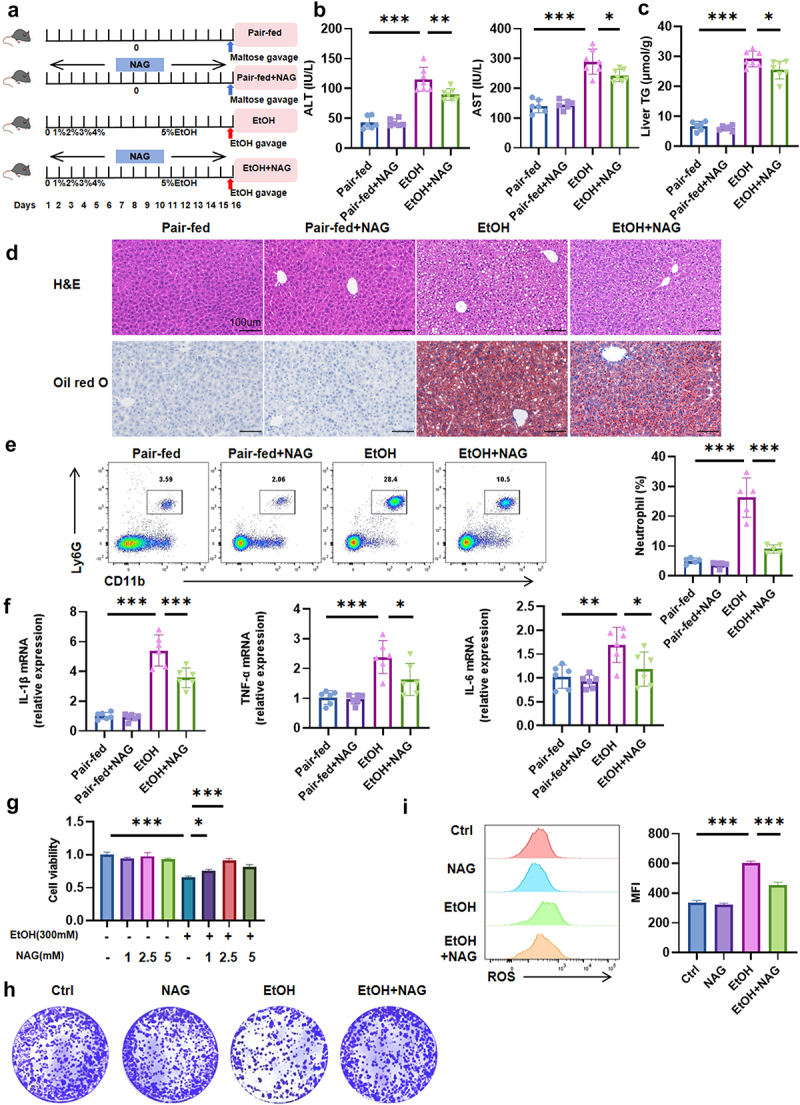
NAG confers profound protection against EtOH-induced liver injury *in vivo* and *in vitro*. (a) Schematic of experimental design. (b) Serum ALT and AST levels. (c) Hepatic TG levels. (d) Representative H&E and Oil red O staining of liver tissues (scale bar: 100 μm). (e) The proportion of neutrophils was analyzed by flow cytometry with statistical graph. (f) Hepatic mRNA expression levels of inflammatory markers *IL-1β*, *TNF-α*, and *IL-6*. (g) Cell viability assay after 48 hours of culture. (h) Cell clonogenic assay. (i) Cellular ROS assay with statistical analysis.

To determine whether the protective effects of *Lachnospiraceae bacterium* in alcohol-associated steatohepatitis are mediated by NAG, we administered exogenous NAG to mice subjected to the NIAAA mouse model (Figure 4(a)). NAG supplementation significantly reduced serum ALT and AST levels, liver TG content, inflammatory cell infiltration, and hepatic steatosis in EtOH-fed mice (Figure 4(b-d)). Additionally, NAG treatment markedly decreased hepatic neutrophil infiltration and downregulated the expression of pro-inflammatory cytokines, including *IL-1β*, *TNF-α*, and *IL-6* (Figure 4(e,f)). The improvements in serum LPS and ZO-1 and Occludin expression following NAG supplementation were consistent with those observed after *Lachnospiraceae bacterium* treatment (Supplementary Figure S4 a, b).

To validate these findings, we conducted *in vitro* experiments using AML12 mouse liver cells. NAG was tested at concentrations of 0, 1, 2.5, and 5 mm to evaluate its safety and efficacy. EtOH exposure (300 mm) significantly inhibited AML12 cell growth, but all tested concentrations of NAG alleviated this inhibition, with the optimal effect observed at 2.5 mm after 48 hours of culture (Figure 4(g), Supplementary Fig. 4C, D). Subsequent experiments utilized these concentrations and culture duration. NAG supplementation not only enhanced AML12 cell proliferation but also significantly reduced reactive oxygen species (ROS) production compared to the EtOH-treated alone group (Figure 4(h,i)). Collectively, these data demonstrate that NAG attenuates EtOH-induced liver damage and oxidative stress in both *in vivo* and *in vitro* models.

### *Either* Lachnospiraceae bacterium *or NAG treatment inhibits ferroptosis during alcohol-associated steatohepatitis*

**Figure 5.**
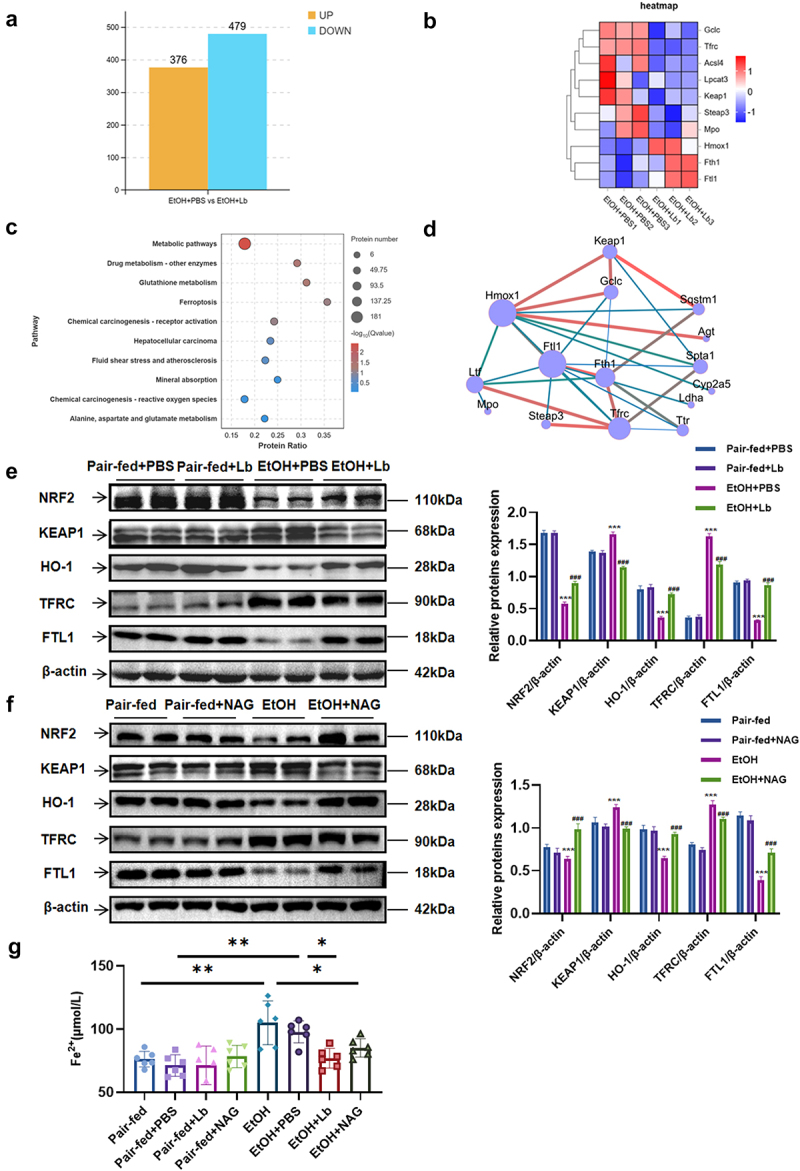
*Lachnospiraceae bacterium* and NAG inhibit ferroptosis in alcohol-associated steatohepatitis. (a) Histogram of differential proteins (VIP > 1, *p* < 0.05) between EtOH and EtOH+Lb groups. (b) Heatmap of differential proteins (VIP > 1, *p* < 0.05) between EtOH and EtOH+Lb groups. (c) KEGG-enriched pathway analysis. (d) PPI network of ferroptosis-related proteins. (e) Protein levels of NRF2, KEAP1, HO-1, TFRC, FTL1, and β-actin in liver tissue with statistical analysis (Pair-fed+PBS vs Pair-fed+Lb vs EtOH+PBS vs EtOH+Lb). (f) Protein levels of NRF2, KEAP1, HO-1, TFRC, FTL1, and β-actin in liver tissue with statistical analysis (Pair-fed vs Pair-fed+NAG vs EtOH vs EtOH+NAG). (g) Hepatic ferrous ion concentrations. *n* = 6–8 per group. *p < 0.05, **p < 0.01, ***p < 0.001. For (E, F): *p < 0.05, **p < 0.01 and ***p < 0.001 vs Pair-fed+PBS/Pair-fed, ^#^*p* < 0.05, ^##^*p* < 0.01, ^###^*p* < 0.001 vs EtOH+PBS/EtOH. Animal model: n = 6–8 per group; Cell model: n = 3 per group. *p < 0.05, **p < 0.01, ***p < 0.001.

To investigate the protective mechanism of *Lachnospiraceae bacterium* against alcohol-associated steatohepatitis in mice, we performed proteomic analysis of liver tissues from EtOH-fed mice, with or without *Lachnospiraceae bacterium* treatment. The analysis identified 376 upregulated and 479 downregulated proteins in the *Lachnospiraceae bacterium*-treated group compared to the controls (Figure 5(a)). Notably, several ferroptosis-related proteins, including ferritin light polypeptide 1 (FTL1), ferritin heavy chain 1 (FTH1), transferrin receptor (TFRC), glutamate-cysteine ligase catalytic subunit (GCLC), lysophosphatidylcholine acyltransferase 3 (LPCAT3), and acyl-CoA synthetase long-chain family member 4 (ACSL4), exhibited significant changes. Additionally, key ferroptosis regulators such as KEAP1 and heme Oxygenase-1 (HO-1) were differentially expressed (Figure 5(b)).

KEGG pathway enrichment analysis highlighted ferroptosis as one of the most significantly altered pathways (Figure 5(c)). Recent studies emphasize the role of the KEAP1-NRF2 pathway in ferroptosis regulation. For instance, apoptotic vesicles from fibroblast-like cells inhibit ferroptosis and promote tissue survival via the KEAP1-NRF2 axis, while ethanolic extracts of *Eclipta prostrata* induce ferroptosis in multiple myeloma through the KEAP1-NRF2/HO-1 axis.^29,30^ Protein-protein interaction (PPI) network analysis further elucidated the interconnections among these differentially expressed proteins (Figure 5(d)).

To validate the proteomic findings, we performed western blotting of key differentially expressed proteins, observing consistency with transcriptional and translational changes (Figure 5E, Supplementary Fig. 5). These results indicate that ferroptosis is inhibited following *Lachnospiraceae bacterium* treatment. We hypothesized that NAG mediates *Lachnospiraceae bacterium-*driven protection against alcohol-associated steatohepatitis by inhibiting ferroptosis. Supporting this, protein and mRNA changes in NAG-treated mice paralleled those observed in *Lachnospiraceae bacterium-*treated mice (Figure 5(f), Supplementary Fig. 6). In addition, ferrous ion assays revealed elevated levels in EtOH-fed mice, which normalized following treatment with either *Lachnospiraceae bacterium* or NAG (Figure 5(g)). These findings demonstrate that both treatments inhibit ferroptosis, potentially through activation of the KEAP1-NRF2 pathway.

### KEAP1-NRF2 pathway is crucial for NAG-exerted hepatoprotection against alcohol-associated steatohepatitis

**Figure 6.**
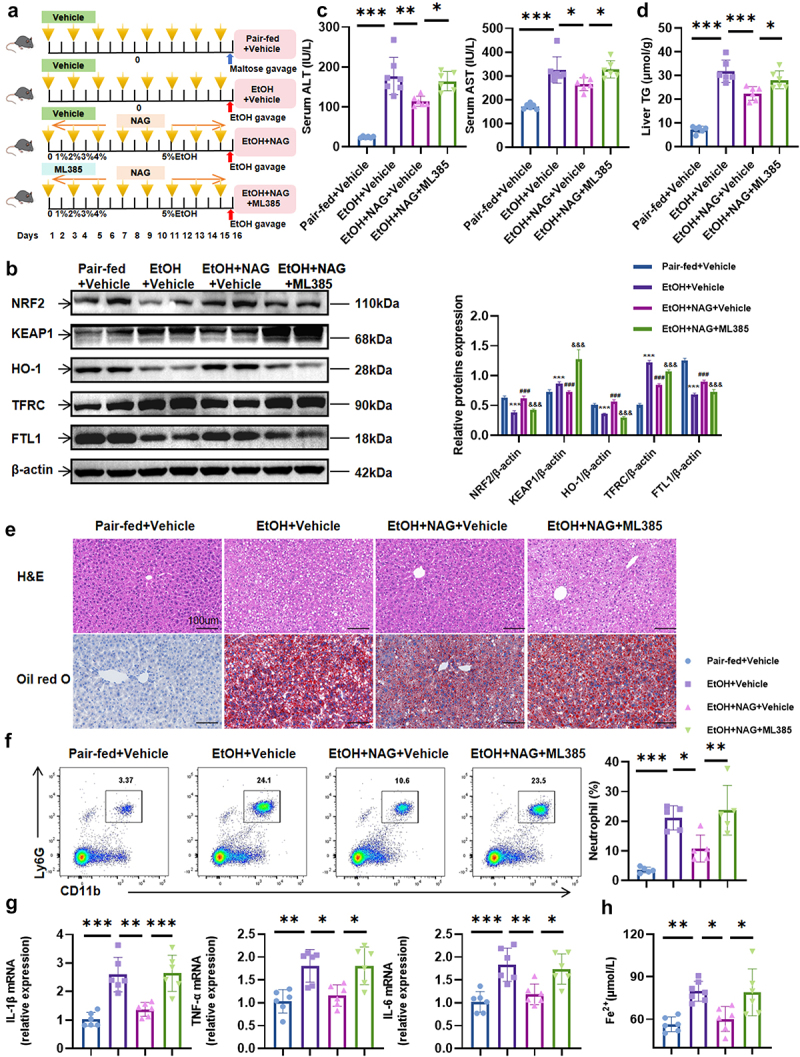
KEAP1-NRF2 pathway mediates NAG-exerted hepatoprotection against alcohol-associated steatohepatitis. (a) Experimental timeline for treatments (Yellow arrows indicate gavage with DMSO or ML385 every other day). (b) Protein levels of NRF2, KEAP1, HO-1, TFRC, FTL1, and β-actin with statistical analysis (Pair-fed+vehicle vs EtOH+Vehicle vs EtOH+NAG+Vehicle vs EtOH+NAG+ML385). (c) Serum ALT and AST levels. (d) Hepatic TG levels. (e) Representative H&E and Oil red O staining of liver tissues (scale bar: 100 μm). (f) The proportion of neutrophils was analyzed by flow cytometry with statistical graph. (g) Hepatic mRNA expression levels of *IL-1β*, *TNF-α*, and *IL-6*. (h) Hepatic ferrous ion concentrations. *n* = 6–8 per group. *p < 0.05, **p < 0.01, ***p < 0.001. For (B): *p < 0.05, **p < 0.01, ***p < 0.001 vs Pair-fed+vehicle, ^#^*p* < 0.05, ^##^*p* < 0.01, ^###^*p* < 0.001 vs EtOH+Vehicle, ^&^*p* < 0.05, ^&&^*p* < 0.01, ^&&&^*p* < 0.001 vs EtOH+NAG+Vehicle.

Our observation that *Lachnospiraceae bacterium* elevates NAG levels, protects against alcohol-associated steatohepatitis, and inhibits ferroptosis through the KEAP1-NRF2 pathway led us to hypothesize that both *Lachnospiraceae bacterium* and NAG exert protection by inhibiting ferroptosis via this pathway. To assess the central role of NRF2, we used the NRF2 inhibitor ML385 (Figure 6(a)) and monitored ferroptosis-related molecules *in vitro* and *in vivo*. As shown in Figure 5, NAG treatment inhibited ferroptosis, evidenced by increased NRF2, HO-1, and FTL1 levels and decreased KEAP1 and TFRC levels. However, ML385 completely abolished NAG’s effects, normalizing protein levels to pre-NAG treatment levels (Figure 6(b), Supplementary Figure7a). Corresponding mRNA changes were also observed (Supplementary Fig.7B, 8). Interestingly, despite KEAP1 being upstream of NRF2, ML385 treatment significantly increased KEAP1 expression, consistent with previous findings.^27,28^

We further evaluated the impact of NRF2 inhibition on NAG-mediated protection against EtOH-induced damage. In EtOH-fed mice, ML385 abrogated NAG’s protective effects, leading to worsened liver injury, increased hepatic steatosis, greater hepatic neutrophil infiltration, and elevated pro-inflammatory cytokine mRNA levels (Figure 6(c-g)). Ferrous ion concentrations significantly increased following ML385 treatment (Figure 6H). Similarly, *in vitro* experiments showed that NRF2 inhibition negated NAG’s protective effect, as demonstrated by reduced cell viability, impaired proliferation, and increased ROS levels (Supplementary Fig.7 C-E). These data confirm that NAG’s hepatoprotective effect against alcohol-associated damage relies on the KEAP1-NRF2 pathway.

## Discussion

ALD has garnered considerable attention due to its potential to be mitigated by modulating the gut microbiota. In this study, we utilized fecal 16S rDNA sequencing and discovered a marked reduction in *Lachnospiraceae bacterium* in fecal samples from ALD patients or EtOH-fed mice compared to healthy controls or pair-fed mice. This reduction is consistent with previous findings,^31,32^ and *Lachnospiraceae bacterium* depletion has been associated with various conditions, including gastric cancer, inflammatory bowel disease, and familial adenomatous polyposis.^33–35^ Zhang et al. demonstrated that the Lachnospiraceae family enhances the immunosurveillance function of CD8+ T cells and controls colorectal cancer progression.^36^ Our study advances these findings by demonstrating a significant negative correlation between *Lachnospiraceae bacterium* abundance and serum ALT, AST, and GGT levels in ALD patients, suggesting a potential protective role for this bacterium in ALD pathogenesis.

The colonization of *Lachnospiraceae bacterium* remodeled the gut microbiota composition, increasing the abundance of beneficial bacteria such as *Akkermansia muciniphila* and *Bacteroides acidifaciens*. *Akkermansia muciniphila*, a gram-negative commensal intestinal bacterium, has well-documented benefits in metabolic and liver diseases, including obesity, diabetes, and ALD.^37–39^ *Bacteroides acidifaciens*, an anaerobic bacterium, has demonstrated protective effects against obesity, colitis,^40,41^ and ALD.^42^ These changes in the gut microbiota composition further substantiate the therapeutic potential of *Lachnospiraceae bacterium* in modulating gut-liver axis dysfunctions in ALD.

To elucidate the protective mechanisms of *Lachnospiraceae bacterium* against alcohol-associated liver injury, we identified N-acetylglutamate (NAG) as a key metabolite associated with the ferroptosis pathway through integrated liver proteomics, fecal metabolomics, and 16S rDNA analysis. Supplementation with NAG significantly alleviated ALD symptoms in mice, indicating its potential therapeutic role. As an intermediate in arginine metabolism, NAG enhances oxidative stress tolerance through epigenetic modifications^43^ and promotes intestinal development and digestion.^44^ Its structural similarity to N-acetylcysteine (NAC), a well-known antioxidant,^45,46^ further highlights its hepatoprotective potential. NAC has been shown to attenuate chronic EtOH-induced hepatic fat accumulation,^47^ modulate autophagy in EtOH-induced hepatocytes,^46^ and reduce cadmium-induced liver fibrosis.^48^ Correlation analyses revealed a positive relationship between *Lachnospiraceae bacterium* abundance and NAG levels, underscoring the role of this bacterium in modulating metabolite production to confer liver protection. This study is the first to demonstrate a significant protective effect of NAG in alcohol-associated steatohepatitis.

Our study further demonstrated that *Lachnospiraceae bacterium* activates the KEAP1-NRF2 pathway, a crucial antioxidant signaling pathway that mitigates oxidative stress and ferroptosis. Chen et al. demonstrated that the traditional Chinese medicinal extract Xiao-Jian-Zhong decoction reduced cadmium-induced hepatic fibrosis via the KEAP1-NRF2 pathway by inhibiting ferroptosis, thus protecting gastric mucosal cells from damage.^49^ Under normal physiological conditions, NRF2 is sequestered in the cytoplasm by KEAP1, which facilitates its ubiquitination and subsequent degradation.^50^ Upon exposure to ROS, KEAP1 is downregulated, impairing NRF2 ubiquitination. This allows NRF2 to translocate to the nucleus, where it induces the expression of various antioxidant factors, including HO-1, γ-glutamylcysteine ligase, and SOD.^51–53^ Treatment with the NRF2 inhibitor ML385 significantly reduced NAG-mediated hepatoprotective effects and exacerbated ferroptosis. These findings suggest that NAG’s protective role is dependent on NRF2 activation. Similarly, NAC has been shown to inhibit ferroptosis in diabetic nephropathy by activating the NRF2 pathway.^54,55^ Consequently, we identified NAG as a small-molecule inducer of the NRF2 pathway, activated by *Lachnospiraceae bacterium* strains.

In conclusion, *Lachnospiraceae bacterium* mitigates ALD by inducing NAG production and activating the KEAP1-NRF2 pathway to inhibit ferroptosis. This study highlights the potential of *Lachnospiraceae bacterium* as a promising probiotic for ALD treatment, with mechanisms involving oxidative stress inhibition, anti-inflammatory effects, and ferroptosis prevention. These findings offer new insights into ALD pathophysiology and open avenues for developing gut microbiota-based therapeutic strategies.

## Supplementary Material

Supplemental Material

## Data Availability

All data in this study are available upon request by contact with the corresponding author.
