# Supplementary material for: Lachnospiraceae bacterium alleviates alcohol-associated liver disease by enhancing N-acetyl-glutamic acid levels and inhibiting ferroptosis through the KEAP1-NRF2 pathway
Source: Gut Microbes. 2025 Jun 13;17(1):2517821. doi: 10.1080/19490976.2025.2517821 (PMC12169036; doi:10.1080/19490976.2025.2517821)
Supplement: Supplemental Material [file KGMI_A_2517821_SM5192.zip › Supplementary materials.docx]

***Lachnospiraceae bacterium*** **alleviates alcohol-associated liver disease by enhancing N-acetyl-glutamic acid levels and inhibiting ferroptosis through the KEAP1-NRF2 pathway**

Hejiao Zhang^1#^, Qiang Hu^1#^, Yong Zhang^2#^, Lei Yang^3^, Shanfei Tian^3^, Xinru Zhang^3^, Haiyuan Shen^4^, Hang Shu^4^, Linxi Xie^3^, Dongqing Wu^4^, Liangliang Zhou^4^, Xiaoli Wei^4^, Chen Cheng^4^, Jiali Jiang^3^, Hua Wang^4,5^, Cailiang Shen^2*^, Derun Kong^1*^, Long Xu^3, 6*^

1 Department of Gastroenterology, the First Affiliated Hospital of Anhui Medical University, Hefei, China;

2 Department of Orthopedics and Spine Surgery, the First Affiliated Hospital of Anhui Medical University, Hefei, China;

3 School of Basic Medical Sciences, Anhui Medical University, Hefei, Anhui, China;

4 Department of Oncology, the First Affiliated Hospital of Anhui Medical University, Hefei, China;

5 Inflammation and Immune Mediated Diseases Laboratory of Anhui Province, Anhui Medical University, Hefei, China;

6 Department of Infectious Diseases, The Second Affiliated Hospital of Anhui Medical University, Hefei, China.

# These authors contribute equally.

***Corresponding authors:**

1. Long Xu, PhD, School of Basic Medical Science, Anhui Medical University, 81 Meishan, Hefei, Anhui, 230032, China. E-mail: xulong@ahmu.edu.cn.

2. Derun Kong, PhD, Department of Gastroenterology, the First Affiliated Hospital of Anhui Medical University, Hefei, Anhui, 230022, China. E-mail: kongderun@ahmu.edu.cn.

3. Cailiang Shen, PhD, Department of Orthopedics and Spine Surgery, the First Affiliated Hospital of Anhui Medical University, Hefei, Anhui, 230022, China. E-mail: shencailiang@ahmu.edu.cn.

**Table 1. Characteristics and liver** **parameters of healthy controls (HC) and patients with alcohol-associated liver disease (ALD)**

| Parameters | HC | ALD |
| --- | --- | --- |
| Sex (male/female) | 20/0 | 20/0 |
| Age (years) | 51.65±1.569 | 50.35±11.70 |
| Body mass index (kg / m ^2^) | 22.13±1.569 | 22.25±1.588 |
| Alcohol consumption (years) | 0 | 22.05±7.577 |
| ALT(U/L) | 13.80±4.797 | 91.40±41.84 |
| AST(U/L) | 17.75±5.056 | 176.1±95.04 |
| AST/ALT | 1.333±0.2563 | 1.875±0.1455 |
| GGT(U/L) | 24.2±5.672 | 286.4±160.0 |

Data are shown as the Mean ± SD.


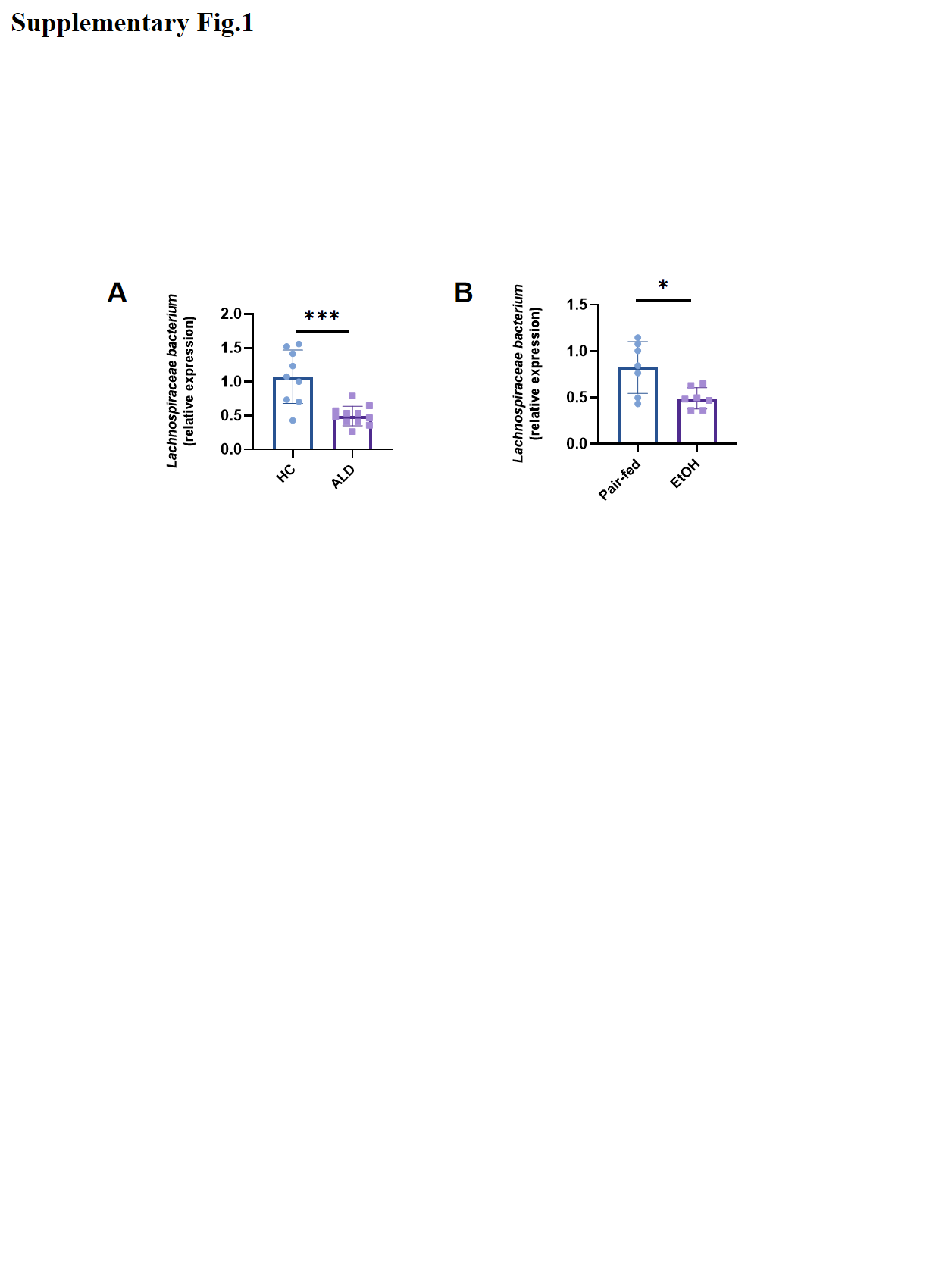


**Supplementary Fig.1 The relative expression of *Lachnospiraceae bacterium* in humans and mice.** (A) The relative expression of *Lachnospiraceae bacterium* in fecal samples from HC and ALD patients was determined by qPCR. (B)The relative expression of *Lachnospiraceae bacterium* in fecal samples from pair-fed and EtOH-fed mice was determined by qPCR. **P* < 0.05, ***P* < 0.01, ****P* < 0.001.


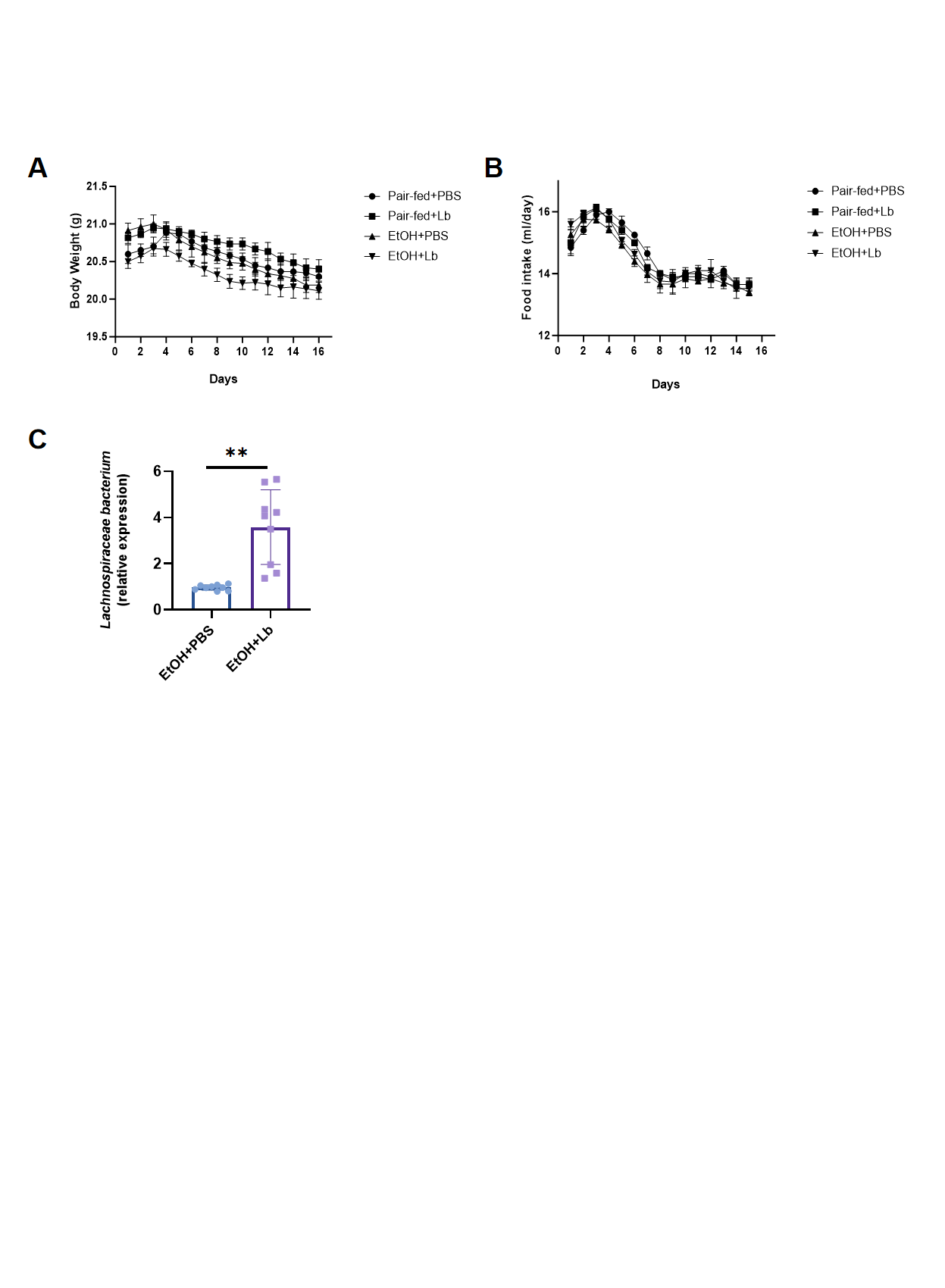


**Supplementary Fig.2 Lack of significant differences in body weight and food intake but successful colonization of *Lachnospiraceae bacterium* in NIAAA model mice.** (A) Daily body weight of mice (g). (B) Daily food intake of mice (mL/day). (C) The relative expression of *Lachnospiraceae bacterium* in fecal samples from EtOH+PBS and EtOH+Lb mice was assessed by qPCR. n = 6-8 per group. ***P* < 0.01.


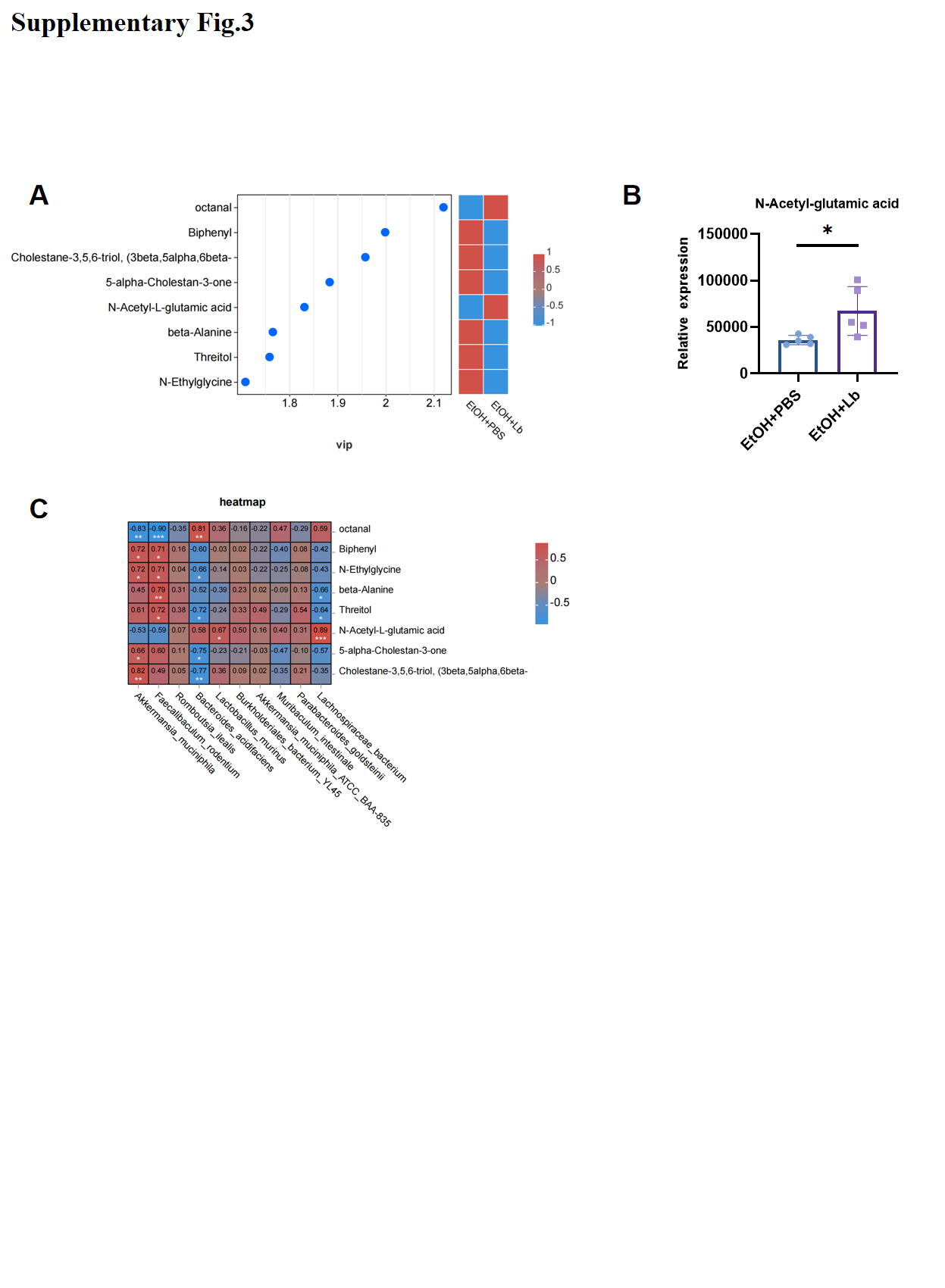


**Supplementary Fig.3 Supplementation with *Lachnospiraceae bacterium* modulates the composition of th****e gut microbiota and significantly increases metabolite NAG.** (A) VIP plot of differential metabolites between the EtOH+PBS group and EtOH+Lb group. (B) Histogram of NAG levels between the EtOH+PBS group and EtOH+Lb group. (C) Heatmap of Spearman's rank correlation coefficient between differential flora and differential metabolites. **P* < 0.05, ***P* < 0.01, ****P* < 0.001.


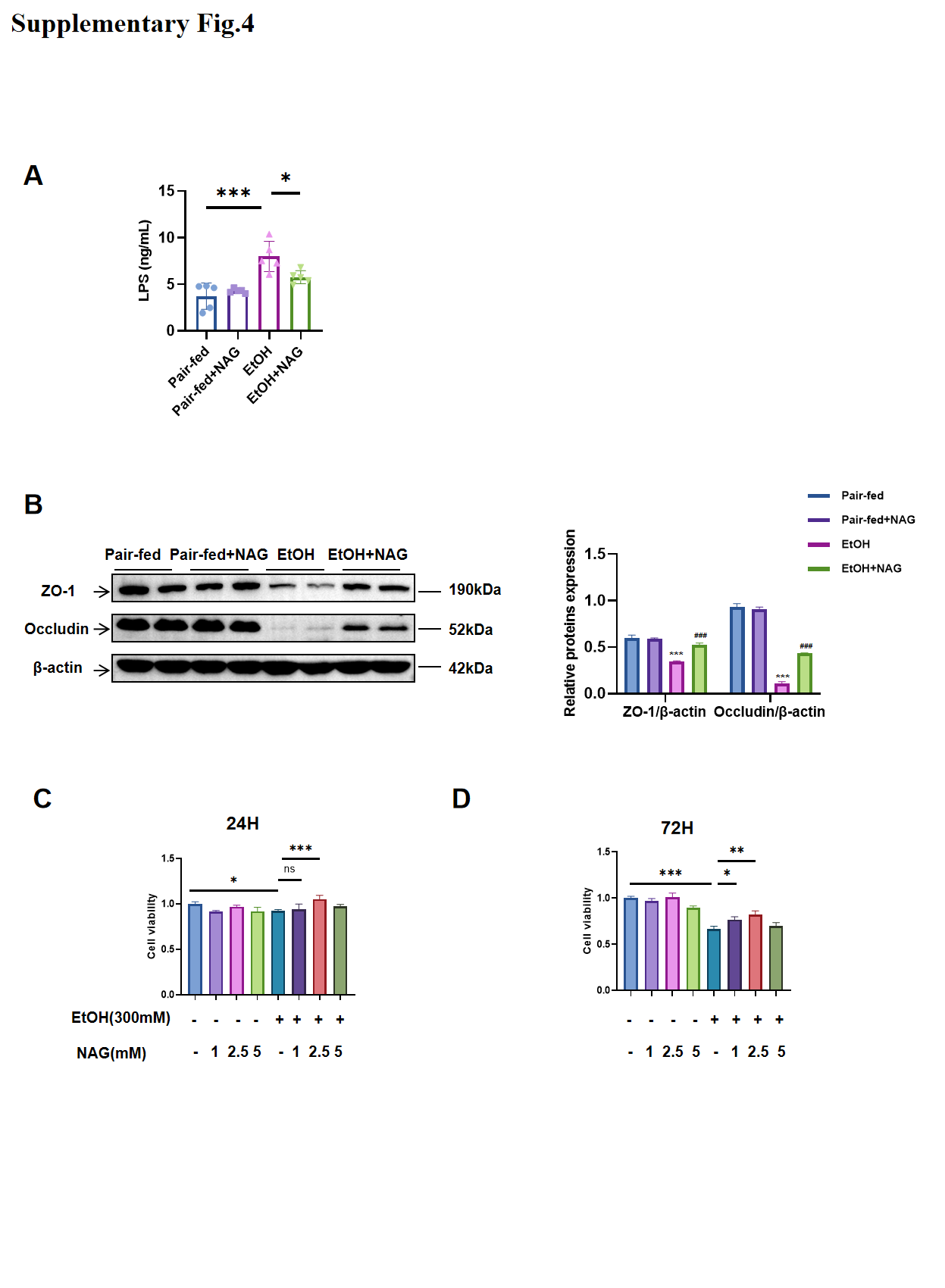


**Supplementary Fig.4 Cell viability, LPS levels, and tight junction protein expression in response to NAG treatment.** (A) Serum LPS levels. (B) Protein expression levels of ZO-1, Occludin, and β-actin in ileum tissue with statistical analysis (Pair-fed vs Pair-fed+NAG vs EtOH vs EtOH+NAG). (C) Cell viability assay after 24 hours of culture. (D) Cell viability assay after 72 hours of culture. n = 5 per group. **P* < 0.05, ***P* < 0.01, ****P* < 0.001. For (B): **P* < 0.05, ***P* < 0.01 and ****P* < 0.001 vs Pair-fed, ^#^*P* < 0.05, ^##^*P* < 0.01, ^###^*P* < 0.001 vs EtOH.


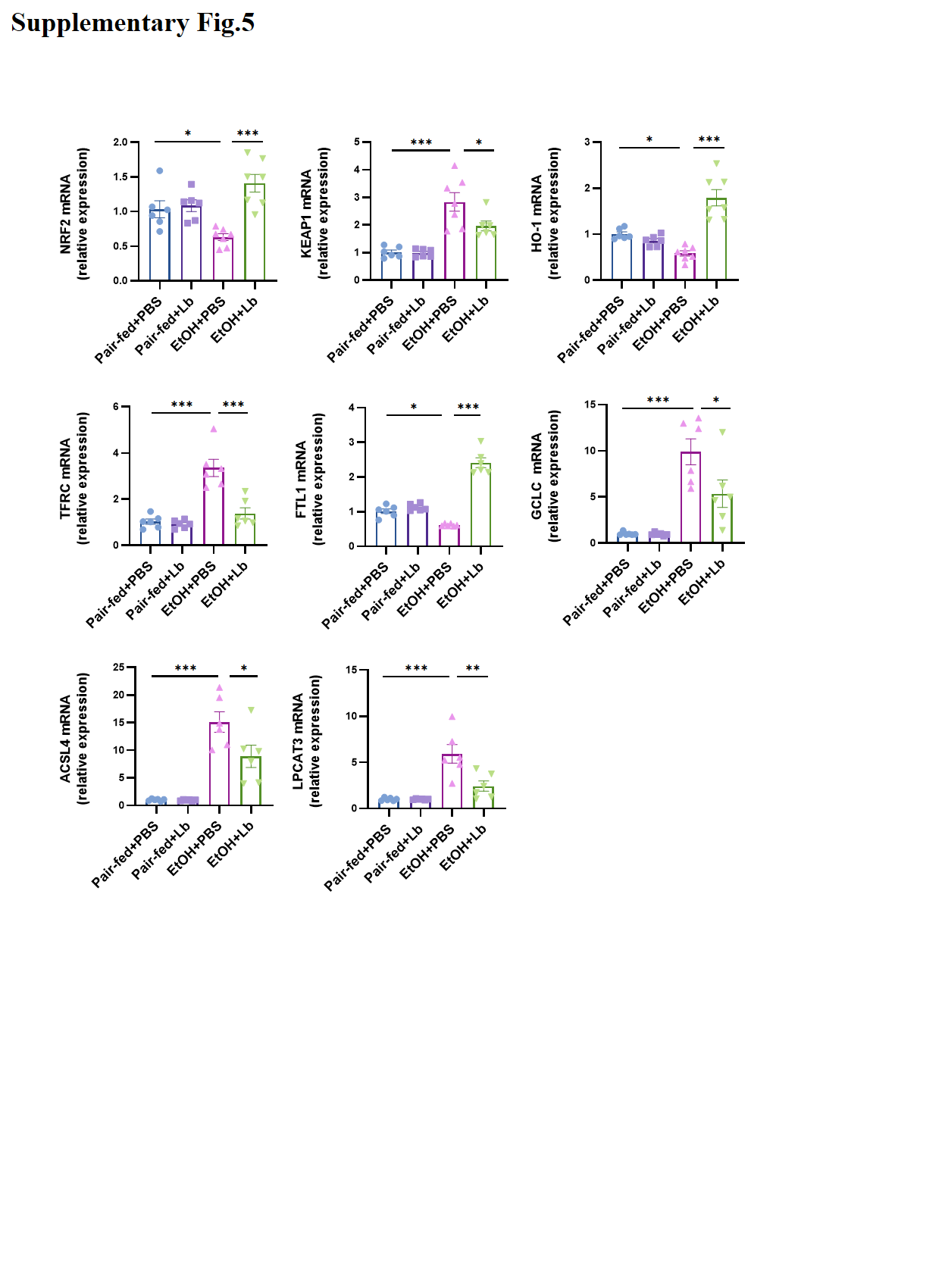


**Supplementary Fig.5 *Lachnospiraceae bacterium* attenuates alcohol-associated steatohepatitis in mice.** mRNA expression levels of *NRF2*, *KEAP1*, *HO-1*, *TFRC*, *FTL1* and *GAPDH* with statistical analysis. n = 6 per group. **P* < 0.05, ***P* < 0.01, ****P* < 0.001.


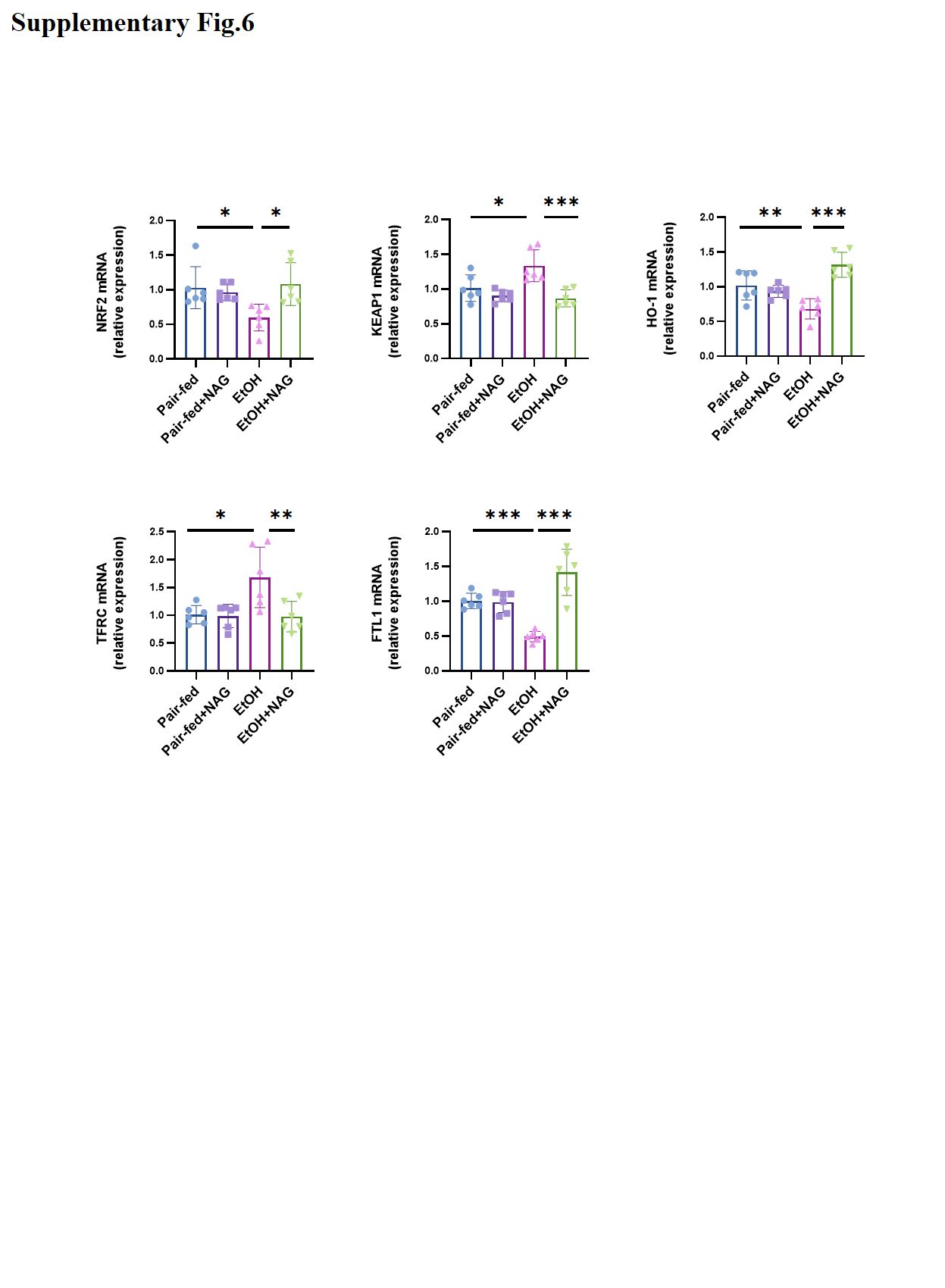


**Supplementary Fig.6 NAG alleviates EtOH-induced damage in AML12 cells.** mRNA expression levels of *NRF2*, *KEAP1*, *HO-1*, *TFRC*, *FTL1* and *GAPDH* with statistical analysis. n = 6 per group. **P* < 0.05, ***P* < 0.01, ****P* < 0.001.


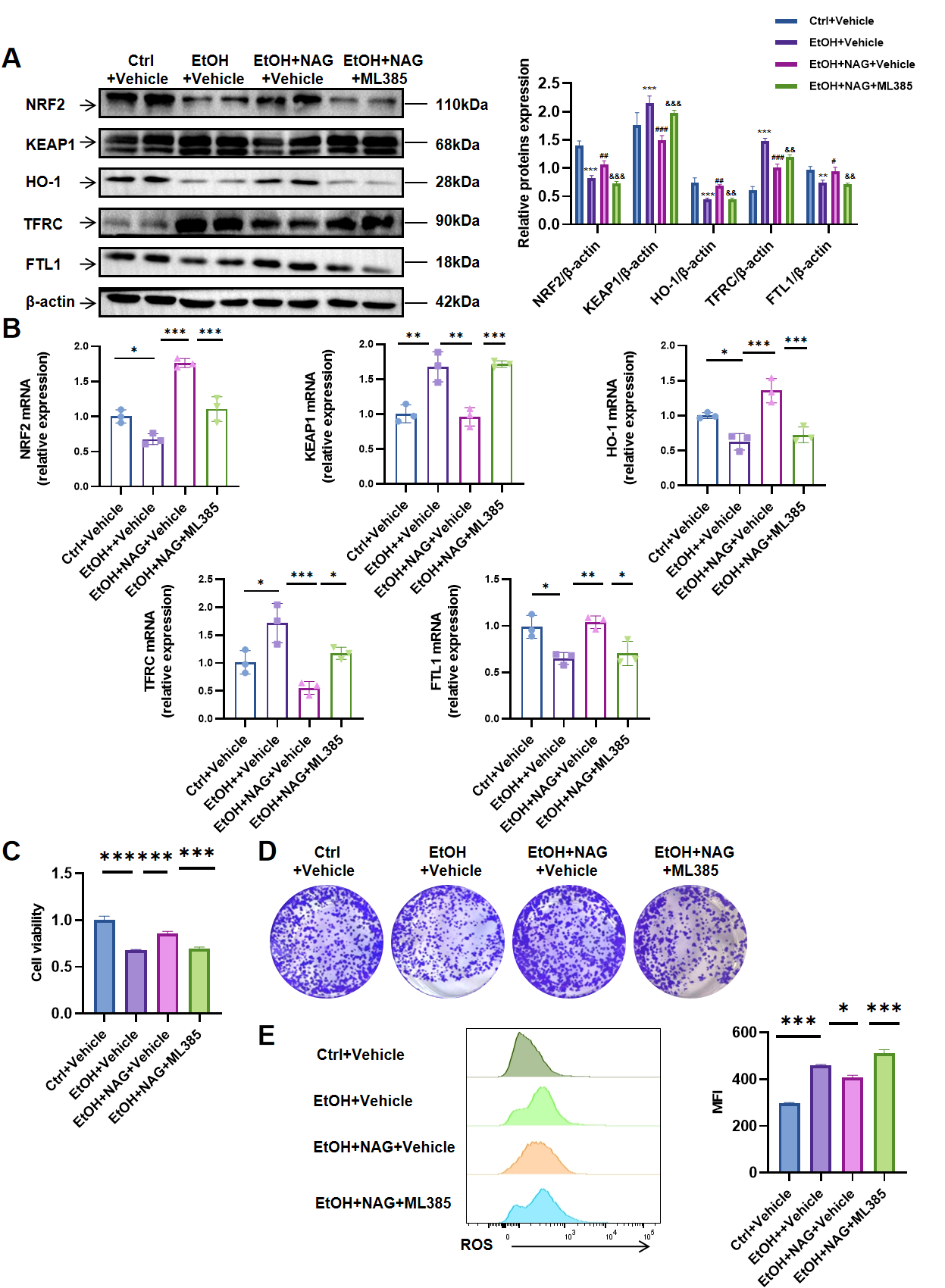


**Supplementary Fig.7 The KEAP1-NRF2 pathway plays an essential role in NAG-exerted hepatoprotection against alcohol exposure** **in AML12 cells.** (A) Protein expression levels of NRF2, KEAP1, HO-1, TFRC, FTL1, and β-actin in AML12 cells with statistical analysis (Ctrl+Vehicle vs EtOH+Vehicle vs EtOH+NAG+Vehicle vs EtOH+NAG+ML385). (B) mRNA expression levels of *NRF2*, *KEAP1*, *HO-1*, *TFRC*, *FTL1* and *GAPDH* with statistical analysis. (C) Cell viability assay. (D) Cell clonogenic assay. (E) Cellular ROS assay with statistical analysis. Cell model: n = 3 per group. **P* < 0.05, ***P* < 0.01, ****P* < 0.001. For (A)： **P* < 0.05, ***P* < 0.01, ****P* < 0.001 vs Ctrl+Vehicle, ^#^*P* < 0.05, ^##^*P* < 0.01, ^###^*P* < 0.001 vs EtOH+Vehicle, ^&^*P* < 0.05, ^&&^*P* < 0.01, ^&&&^*P* < 0.001 vs EtOH+NAG+Vehicle.


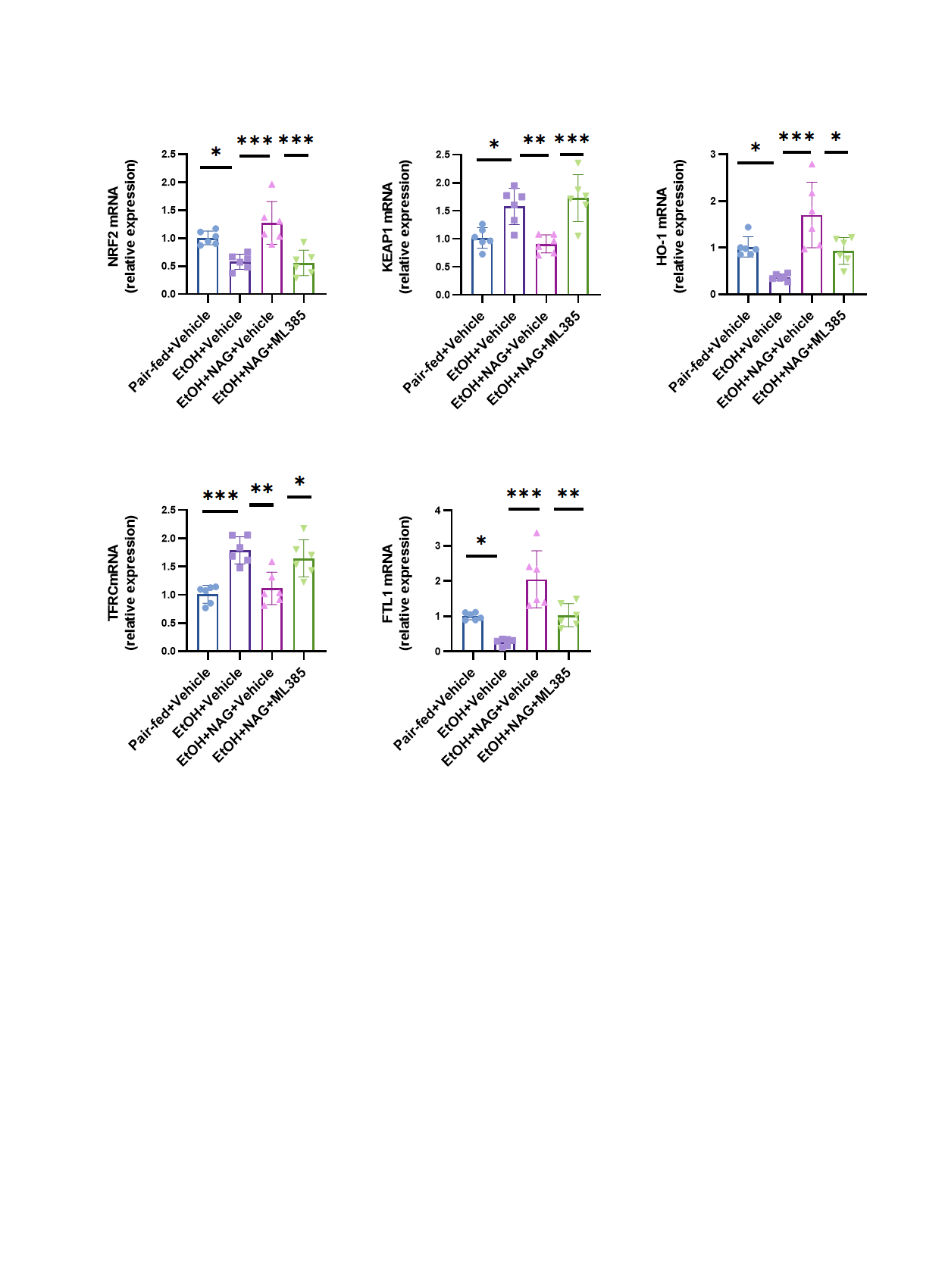
**Supplementary Fig.8 KEAP1-NRF2 pathway plays an essential role in NAG-exerted hepatoprotection against alcohol-associated steatohepatitis in mice.** mRNA expression levels of *NRF2*, *KEAP1*, *HO-1*, *TFRC*, *FTL1* and *GAPDH* with statistical analysis. n = 6 per group. **P* < 0.05, ***P* < 0.01, ****P* < 0.001.

**Table 2. Sequences of primers for qPCR**

| Genes name | Forward primer | Reverse primer |
| --- | --- | --- |
| *GAPDH* | AGCAGCCGCATCTTCTTGTGCAGTG | GGCCTTGACTGTGCCGTTGAATTT |
| *IL-1β* | TCGCTCAGGGTCACAAGAAA | CATCAGAGGCAAGGAGGAAAAC |
| *TNF-α* | AGGCTGCCCCGACTACGT | GACTTTCTCCTGGTATGAGATAGCAAA |
| *IL-6* | TCCATCCAGTTGCCTTCTTG | TTCCACGATTTCCCAGAGAAC |
| *KEAP1* | GATATGAGCCAGAGCGGGAC | CATACAGCAAGCGGTTGAGC |
| *NRF2* | AAAATCATTAACCTCCCTGTTGAT | CGGCGACTTTATTCTTACCTCTC |
| *HMOX1* | CAAGCCGAGAATGCTGAGTTCATG | CAAGCCGAGAATGCTGAGTTCATG |
| *FTL1* | ATGGGCAACCATCTGACCAA | TTGAGAGTGAGGCGCTCAAA |
| *TFRC* | AAGTGACGTAGATCCAGAGGG | GACAATGGTTCCCCACCAAA |
| *GCLC* | CTGCACATCTACCACGCAGT | TTCATGATCGAAGGACACCA |
| *ACSL4* | CCTTTGGCTCATGTGCTGGAACT | CAGCGGCCATAAGTGTGGGTTT |
| *LPCAT3* | GGCCTCTCAATTGCTTATTTCA | AGCACGACACATAGCAAGGA |
| *16s* | CCGTCAATTCMTTTGAGTTT | ACTCCTACGGGAGGCAGCAG |
| *Lb* | AGAGTTTGATCCTGGCTCAG | GGTTACCTTGTTACGACTT |
