# Supplementary material for: Lachnospiraceae bacterium alleviates alcohol-associated liver disease by enhancing N-acetyl-glutamic acid levels and inhibiting ferroptosis through the KEAP1-NRF2 pathway
Source: Gut Microbes. 2025 Jun 13;17(1):2517821. doi: 10.1080/19490976.2025.2517821 (PMC12169036; doi:10.1080/19490976.2025.2517821)
Supplement: Supplemental Material [file KGMI_A_2517821_SM5192.zip › Original_Western_blots.docx]

# ZO-1


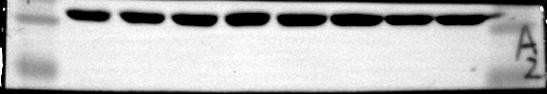

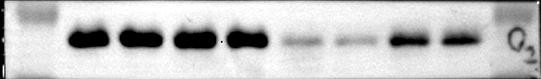

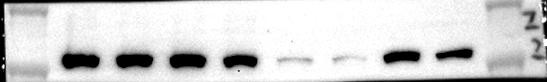


**Occludin β-actin**

**250kDa 180kDa 60kDa**

**50kDa 40kDa 30kDa**

# TFRC

**β-actin HO-1 FTL1**

**180kDa**

130kDa


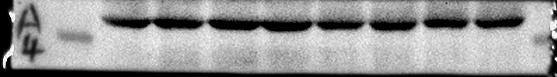

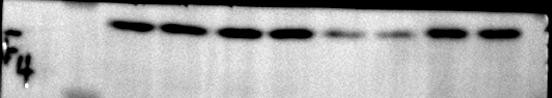

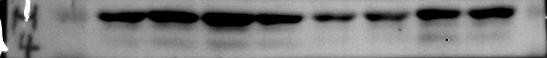

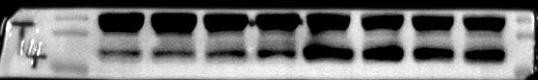


100kDa 40kDa 35kDa

25kDa 15kDa

# NRF2 KEAP1

**β-actin**

130kDa

100kDa 70kDa


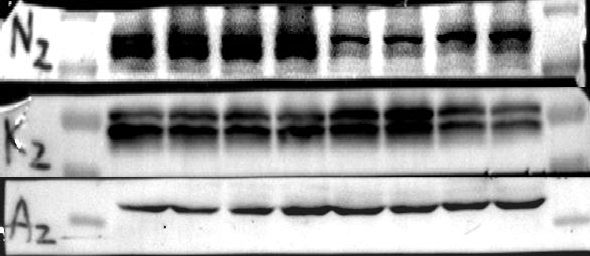


55kDa 40kDa

# TFRC KEAP1

**β-actin**

# HO-1

**NRF2**

**β-actin FTL1**

**180kDa**

130kDa


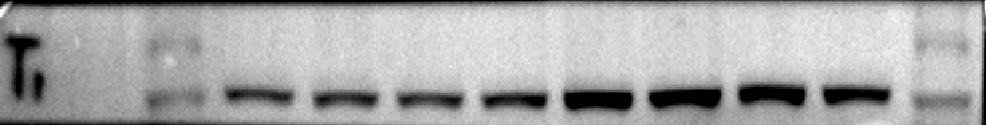

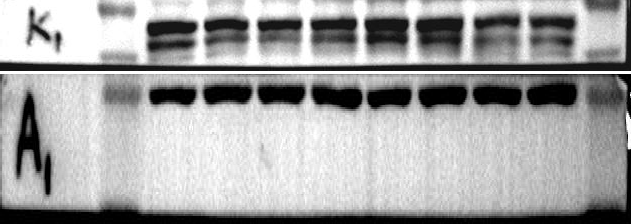

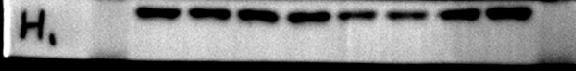


100kDa 70kDa

55kDa 40kDa

35kDa

25kDa

180kDa 130kDa


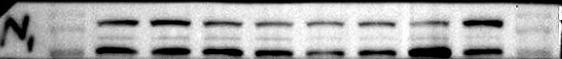

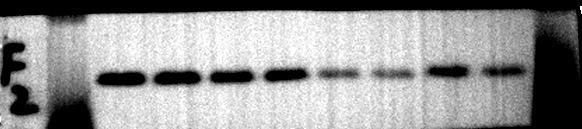

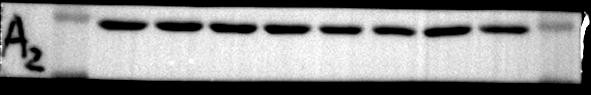


100kDa 40kDa

35kDa 25kDa

15kDa

# TFRC KEAP1

**β-actin**

# HO-1 FTL1

**NRF2**

**β-actin**

**180kDa**

130kDa 100kDa 70kDa


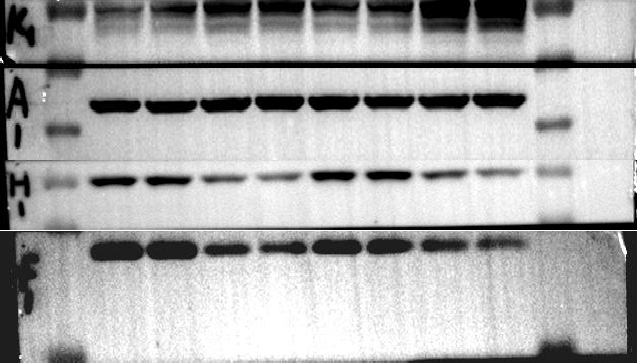

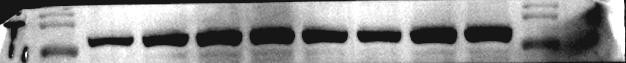


55kDa 40kDa 35kDa

25kDa

15kDa

180kDa 130kDa 100kDa


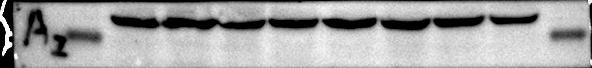

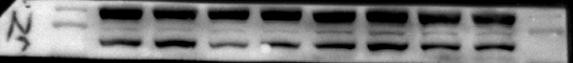


40kDa 35kDa

# ZO-1


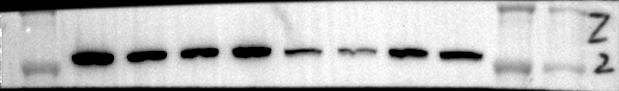


**Occludin β-actin**


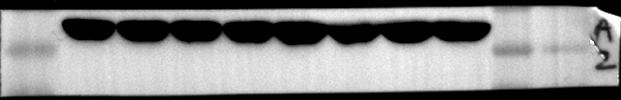

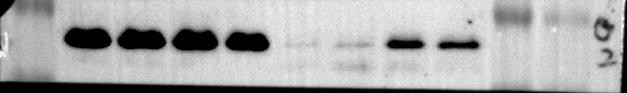


**250kDa 180kDa 60kDa 50kDa 40kDa**

# NRF2

**β-actin**

180kDa 130kDa

100kDa 40kDa


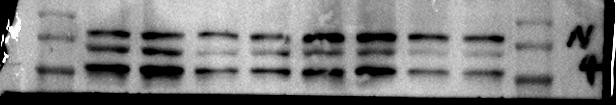

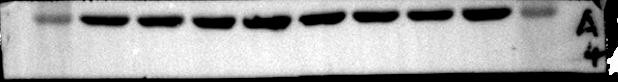


35kDa

# KEAP1

**β-actin HO-1**

# FTL1

**TFRC**

**β-actin**

70kDa 55kDa 40kDa 35kDa

25kDa 15kDa


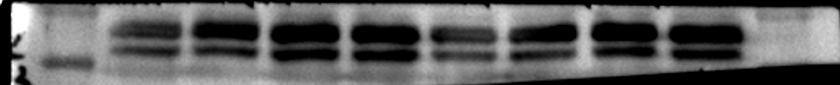

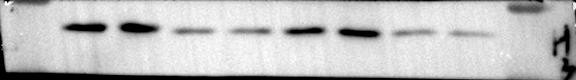

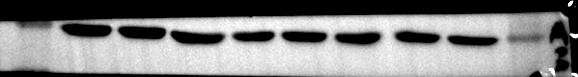

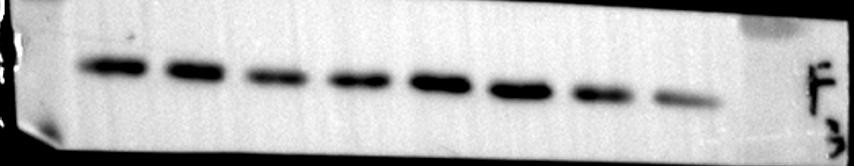


**180kDa**


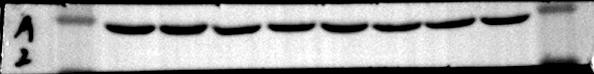

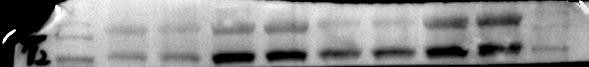


130kDa

100kDa 40kDa

35kDa
